# Mutation of a single residue promotes gating of vertebrate and invertebrate two-pore domain potassium channels

**DOI:** 10.1038/s41467-019-08710-3

**Published:** 2019-02-15

**Authors:** Ismail Ben Soussia, Sonia El Mouridi, Dawon Kang, Alice Leclercq-Blondel, Lamyaa Khoubza, Philippe Tardy, Nora Zariohi, Marie Gendrel, Florian Lesage, Eun-Jin Kim, Delphine Bichet, Olga Andrini, Thomas Boulin

**Affiliations:** 1 Institut NeuroMyoGène, Univ Lyon, Université Claude Bernard Lyon 1, CNRS UMR 5310, INSERM U1217, Lyon, 69008 France; 2 Department of Physiology, College of Medicine and Institute of Health Sciences, Gyeongsang National University, Jinju, 52727 South Korea; 3 Institut de Pharmacologie Moléculaire et Cellulaire, LabEx ICST, CNRS UMR 7275, Université de Nice Sophia Antipolis, Valbonne, 06560 France

## Abstract

Mutations that modulate the activity of ion channels are essential tools to understand the biophysical determinants that control their gating. Here, we reveal the conserved role played by a single amino acid position (TM2.6) located in the second transmembrane domain of two-pore domain potassium (K2P) channels. Mutations of TM2.6 to aspartate or asparagine increase channel activity for all vertebrate K2P channels. Using two-electrode voltage-clamp and single-channel recording techniques, we find that mutation of TM2.6 promotes channel gating via the selectivity filter gate and increases single channel open probability. Furthermore, channel gating can be progressively tuned by using different amino acid substitutions. Finally, we show that the role of TM2.6 was conserved during evolution by rationally designing gain-of-function mutations in four *Caenorhabditis elegans* K2P channels using CRISPR/*Cas9* gene editing. This study thus describes a simple and powerful strategy to systematically manipulate the activity of an entire family of potassium channels.

## Abstract (web summary)

Mutations that modulate the activity of ion channels are essential tools to understand the biophysical determinants that control their gating. Here authors reveal the role played by a single residue in the second transmembrane domain of vertebrate and invertebrate two-pore domain potassium channels.

## Introduction

Two-pore domain potassium (K2P) channels play a central role in the regulation of cellular excitability and the establishment of the membrane potential in excitable and non-excitable cells^1^. This ancient ion channel family has been widely conserved during evolution. Genes encoding K2P channel subunits are found in the genomes of yeast, plants, vertebrates, and invertebrates. Fifteen and eleven genes encoding channel subunits are found in the human and *Drosophila melanogaster* genomes, respectively^2^. Strikingly, a large expansion of the K2P channel gene family has occurred in the model nematode *Caenorhabditis elegans*^3^. While the overall number of genes encoding potassium channel subunits does not differ significantly (approx. 80 in man vs. 70 in *C*. *elegans)*, more than half of these genes (47) encode two-pore domain potassium channel subunits. Interestingly, while all K2P channels share a characteristic topology and domain structure, sequence conservation at the amino acid level is generally low except for closely-related paralogs. Therefore, analyzing sequence diversity in vertebrate and invertebrate K2P channels can offer interesting insights into the key molecular determinants that make up the functional core of this large ion channel family.

Studies aiming to dissect the gating mechanisms of K2P channels have relied on the identification of residues that modify channel activity when mutated. Yeast selection assays^4^, targeted mutagenesis^5–11^, and genetic screens^5,6^ have highlighted different amino acid positions that increase channel activity for various K2P channels. Pathogenic mutations have also revealed residues such as the TALK2 G88R mutation which increases channel function and causes a severe and progressive cardiac conduction disorder^7^. Unfortunately, since these mutations affect residues situated in very different parts of the channel (transmembrane domains, pore loops, extracellular, and cytoplasmic regions) and are rarely conserved in all K2P channels, no single residue has so far emerged that could play a conserved role in the control of K2P channel gating.

Interestingly though, we and others have shown that mutations in one position of the second transmembrane segment (further referred to as TM2.6, see results below) have consistent effects in three vertebrate and one invertebrate channel. First, mutation of a glycine residue in Drosophila KCNK0/ORK1 was serendipitously found to maximize channel open probability^8^. Next, mutation of a leucine residue at the equivalent position was shown to increase the activity of TWIK1^9^. Finally, mutation of isoleucine in THIK1 and THIK2 similarly increased whole-cell current levels^10^. Based on crystal structures and sequence alignments, these four residues are positioned on the intracellular face of the second transmembrane domain, exposed to the cytoplasmic vestibule, and in close proximity with the intracellular part of the selectivity filter^12–15^. Although the exact mechanism by which these mutations increase channel activity remains to be fully understood, this position nevertheless appeared as a promising candidate for a residue that could play a conserved role in most if not all K2P channels.

In this study, we have combined two-electrode voltage-clamp electrophysiology in *Xenopus* oocytes, single-channel recording in cultured cells, and CRISPR/*Cas9* gene editing in *C*. *elegans* to demonstrate the functional conservation of TM2.6 in vertebrate and invertebrate two-pore domain potassium channels. We show that all vertebrate K2Ps can be activated by mutating TM2.6. These mutations dramatically increase channel activity and in particular single-channel open probability. Consistently, mutating the homologous residue in four *C*. *elegans* K2P channels elicited behavioral phenotypes that were comparable to those of known gain-of-function mutants. Finally, by building allelic series in *C*. *elegans* and for channels expressed in *Xenopus* oocytes, we could further demonstrate that channel activity can be progressively tuned by using different TM2.6 amino acid substitutions. Taken together, these results demonstrate that the TM2.6 position plays an important and well-conserved role in the gating of many if not all two-pore domain potassium channels.

## Results

### Sequence conservation in distantly-related K2P channels

Two-pore domain potassium (K2P) channels were first identified in the genomes of yeast and *C*. *elegans* based on their characteristic structure as tandems of pore-forming domains^11^. Interestingly, while all K2P channels share this basic topology, their amino acid sequences have diverged markedly. Vertebrate K2P channels have been classified in 6 families based on their peptide sequences and functional properties. Only members of a given class share high levels of homology (Supplementary Fig. 1). Sequence variation is even more striking for *C*. *elegans* K2P channels. Within the 47 genes encoding subunits of K2P channels, sequence conservation is generally low except for close relatives. Only five *C*. *elegans* channels exhibit significant sequence identity with vertebrate orthologs. SUP-9 and TWK-20 are most similar to TASK1/3/5, TWK-14 is a clear ortholog of THIK1/2, and TWK-46 and TWK-48 resemble TWIK1/2 and TRESK, respectively (Supplementary Fig. 1). For most other nematode K2Ps, on average 25% of amino acids are identical to the closest vertebrate relative, even when only the core portion of the channel—from the first to the fourth transmembrane segment—is considered, and variable N- and C-terminal cytoplasmic regions are omitted. For a few *C*. *elegans* K2P channels, conservation with vertebrate channels is even lower, ranging from 16% to 25% (*twk-6*, *twk-29*, *twk-32*, *twk-47*), although bioinformatic analyses suggest that they retain the characteristic domain organization.

**Fig. 1:**
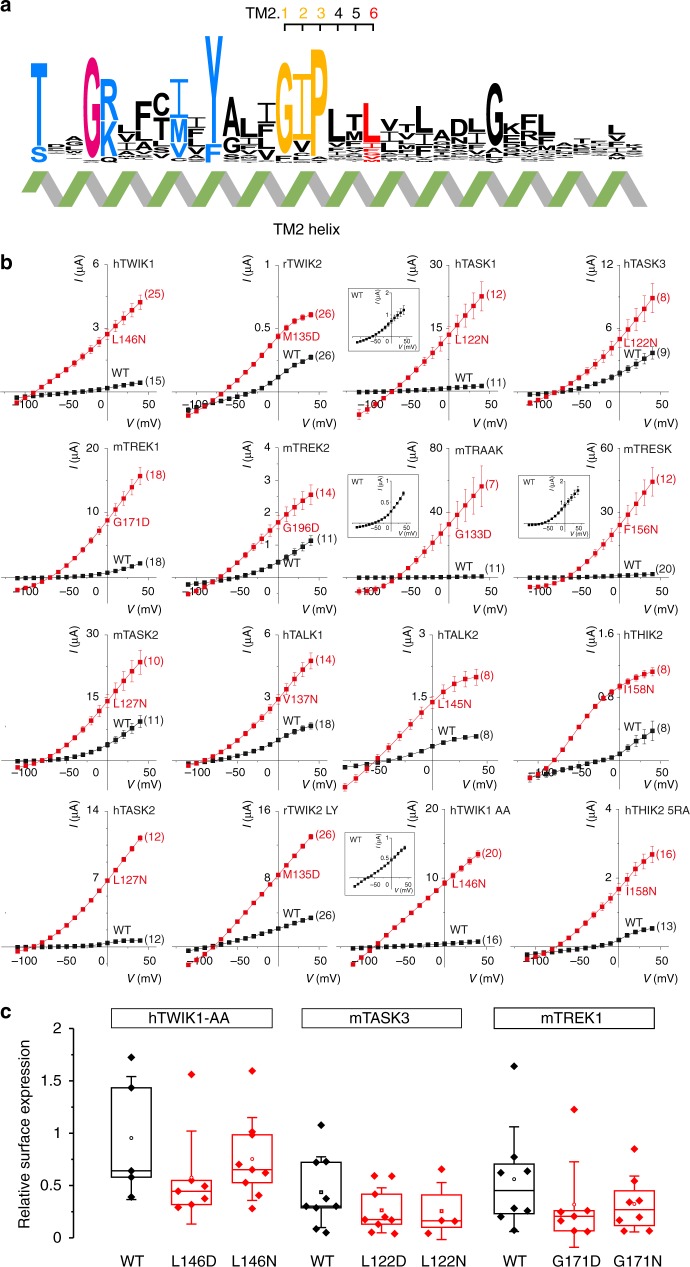
Mutation of a single residue in TM2 systematically increases K2P channel activity. **a** Sequence conservation along transmembrane helix 2 (TM2 helix) computed from 66 vertebrate, insect, and nematode K2P channels, represented using WebLogo 3 ^53^. **b** Current–voltage relationships obtained at pH 7.4 in *X*. *laevis* oocytes by injection of cRNA encoding wild-type (black squares) and TM2.6 mutant channels (red squares). TM2.6 mutations are indicated in red next to corresponding current traces. Insets for hTASK1, mTRAAK, mTRESK, and hTWIK1 AA represent wild-type channel currents at a reduced scale. rTWIK2 LY and hTHIK2 5RA harbor additional mutations in intracellular trafficking signals that allow increased surface expression^10,52^. Each point represents the mean ± standard error of the mean, numbers in parentheses represent the number of oocytes tested for each condition. Injected cRNA amounts and incubation times are reported in Supplementary Table 1. **c** Relative surface expression for wild-type and TM2.6 mutant channels using flow cytometry. HA/GFP-tagged wild-type (black) and mutant K2P channels (red) were expressed in HEK cells. Relative surface expression was determined by measuring the median fluorescence intensity of HA-positive cells (labeled in non-permeabilizing conditions) relative to the median fluorescence intensity of the GFP signal (total channel content). Center lines, medians; open circles, means; box limits, 25th and 75th percentiles; whiskers, standard deviation. Kruskal–Wallis, Dunn’s multiple comparison test, *p* > 0.05

We took advantage of this long evolutionary history to identify residues that could be important for the structure and function of K2P channels. Using an alignment of 66 vertebrate and invertebrate channels, we identified highly conserved residues in three transmembrane helices (TM1, TM2, TM4), two pore helices (Ph1, Ph2), and the selectivity filter (SF1, SF2) sequences (Fig. 1a, Supplementary Fig. 2). In contrast, the sequence of the third transmembrane helix (TM3) did not appear to be under selective pressure.

We next focused on the second transmembrane domain (TM2), and in particular on its central region because it contains the leucine and glycine residues which activate TWIK1, THIK1/2, and ORK1/KCNK0 channels when mutated^8–10^. As in other potassium channel families, conserved glycine residues are found at the center of TM2 in almost all K2Ps (Supplementary Fig. 2). They provide flexibility to these large membrane-spanning helices allowing for the conformational changes thought to be involved in channel gating^16–18^. In addition, the central region of TM2 presented remarkable sequence conservation in distant K2P channels (Fig. 1a). In 48 of 66 channels, this glycine hinge is followed by isoleucine and proline residues (further referred to as the GIP motif). In TWIK2, TASK2, Drosophila CG1688, and three *C*. *elegans* channels (TWK-9, TWK-20, and TWK-40), the isoleucine is replaced by a valine. Mammalian THIK1/2 and *C*. *elegans* TWK-14 channels have a different motif in which isoleucine is replaced by cysteine, and proline by one of three amino acids (serine, alanine or threonine). Further degenerate motifs are found in Drosophila *Sandman* (GMP), nine *C*. *elegans* channels (MIP, FVP, GIA, GLP, GAP), and the silent channel KCNK7 (GLP).

Sequence conservation of the residues following the GIP motif (and its derivatives) is less obvious in vertebrates. However, a common theme can be observed when 8 Drosophila and 43 *C. elegans* channels are included in the analysis. In particular, the third residue following the GIP motif is generally a leucine (44/66) or an isoleucine (6/66), and more rarely, a glycine (TREK1, TREK2, TRAAK, KCNK0/ORK1), a phenylalanine (TRESK, TWK-17, TWK-24, UNC-58), a valine (TALK1, TWK-14, TWK-18, TWK-29), a methionine (TWIK2, TWK-16, TWK-43), or an alanine (TWK-25). To facilitate the identification of homologous residues in distantly-related K2P channels, we introduce a nomenclature to identify residues in TM2 by using the conserved glycine hinges as reference positions. This glycine is labeled TM2.1 and the subsequent isoleucine and proline residues of the GIP motif are referred to as TM2.2 and TM2.3. The next three residues are labeled TM2.4, TM2.5, and TM2.6 (Fig. 1a). The remainder of this study will describe the conserved role played by TM2.6 in the control of K2P channel gating.

### TM2.6 mutation increases activity of vertebrate K2P channels

To test whether TM2.6 mutation could systematically increase the activity of vertebrate K2Ps, we generated point mutations of TM2.6 to asparagine or aspartate in 16 different rodent (r, *Rattus norvegicus*; m, *Mus musculus*) and human K2P channels (Fig. 1b). We then used two-electrode voltage-clamp recording in *Xenopus* oocytes to compare the total current elicited by wild-type and mutant channels. We found that in all cases, currents were markedly increased for TM2.6 mutants and their selectivity for potassium was maintained as indicated by marked hyperpolarization of reversal potentials (Supplementary Table 1). Depending on the channel, we observed 3- to more than 100-fold increases in total current.

To test whether this increase in current could be simply explained by an increase in surface expression of the channels, we generated hTWIK1-AA, mTASK3, and mTREK1 channels harboring HA-epitope tags in their extracellular Ph2 to M4 loops, and C-terminal GFP fluorescent proteins. We then used immunofluorescence in non-permeabilizing conditions combined with flow cytometry to compare levels of surface expression. In brief, HEK-293 cells were transfected with HA/GFP-tagged wild-type or TM2.6 mutant channels, labeled with primary anti-HA and secondary Alexa594-conjugated antibodies in non-permeabilizing conditions, and sorted by flow cytometry based on two-color fluorescence. Median fluorescence intensity (MFI) was determined for HA and GFP signals and used to determine the relative surface expression. These flow cytometry experiments did not show increased expression of TM2.6 mutant vs. wild-type channels (Fig. 1c), therefore demonstrating that the current increase observed for TM2.6 mutants did not simply result from increased surface expression, but more likely resulted from direct effects on the intrinsic activity of K2P channels.

Taken together, these experiments provide evidence for the consistent role played by TM2.6 in the modulation of channel activity for all vertebrate two-pore domain potassium channels.

### TM2.6 mutation promotes gating of pH-sensitive K2P channels

**Fig. 2:**
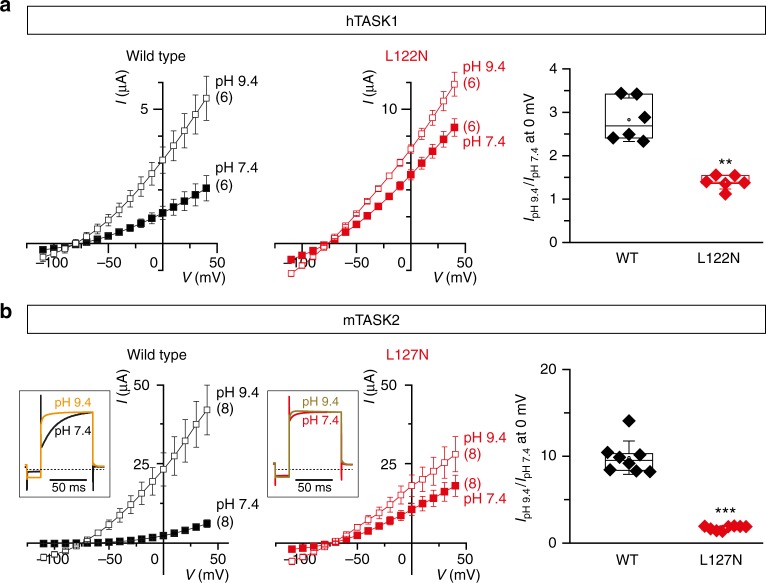
TM2.6 mutation increases the basal activity of pH-sensitive K2P channels. Current–voltage relationships of **a** hTASK1 and **b** mTASK2 channels; wild-type (black) and TM2.6 mutant (red) at extracellular pH 7.4 (filled squares) and pH 9.4 (open squares). Each data point represents the mean ± standard error of the mean, numbers in parentheses represent the number of oocytes tested for each condition. In (**b**), insets represent normalized current traces recorded at 0 mV. mTASK2 activation time was 91.07 ± 2.27 ms (*n* = 8) at pH 7.4 (black trace) and 44.98 ± 4.96 ms (*n* = 8) at pH 9.4 (orange trace). mTASK2 L127N activation time was 39.51 ± 2.25 ms (*n* = 8) at pH 7.4 (red trace) and 29.41 ± 5.35 ms (*n* = 5) at pH 9.4 (brown trace). Rightmost panels represent relative current increases, corresponding to the ratio between currents under pH 9.4 perfusion and currents under pH 7.4 perfusion at 0 mV. Mean relative current increases at 0 mV from pH 7.4 to 9.4 were 2.8 ± 0.2 and 1.4 ± 0.1 for wild-type and mutant hTASK1, respectively. Mean relative current increases were 9.8 ± 0.7 and 1.8 ± 0.1 for wild-type and mutant mTASK2, respectively. Currents were recorded 24 h after injection of cRNA (mTASK2 and mTASK2 L127N, 5 ng/oocyte; hTASK1, 2.28 ng/oocyte; hTASK1 L122N, 0.38 ng/oocyte). Each data point represents one oocyte; center lines, medians; open circles, means; box limits, 25th and 75th percentiles; whiskers, standard deviation. Mann–Whitney, **p < 0.01, ***p < 0.001

To characterize the impact of TM2.6 mutations on channel function, we analyzed the responses of TASK1 and TASK2 channels to changes in extracellular pH. Indeed, alkalinization of the extracellular medium increases the activity of these channels via a direct effect on the selectivity filter (or C-type) gate. Specifically, raising extracellular pH promotes the open state of TASK1^12^, and increases the opening frequency of TASK2^13–15^. We reasoned that if mutant channels were already highly active at physiological pH, they should be less sensitive to stimulation by extracellular alkalinization. Indeed, raising extracellular pH from 7.4 to 9.4 only increased whole-cell currents of TASK1 L122N by a factor of 1.4, while wild-type currents increased 2.8-fold (Fig. 2a). Similarly, TASK2 L127N currents were only increased by a factor of 2, while pH challenge led to a 10-fold increase in total current for wild-type TASK2 (Fig. 2b).

Furthermore, we found that the activation kinetics of TASK2 channels were markedly affected. Expression of wild-type TASK2 in *Xenopus laevis* oocytes gives rise to a slowly activating outward current in response to depolarizing voltage steps at physiological pH^13,19^. The activation kinetics of wild-type TASK2 increase sharply when TASK2 is stimulated by alkaline pH. Consistent with high basal activity, TASK2 L127N no longer showed the slow time-dependent outward current at neutral pH (Fig. 2b, inset).

Taken together, these experiments suggest that TM2.6 mutants of TASK1 and TASK2 are significantly more active in physiological conditions, likely due to increased activation of the selectivity filter gate.

### TM2.6 mutation increases activity of mechano-gated K2Ps

**Fig. 3:**
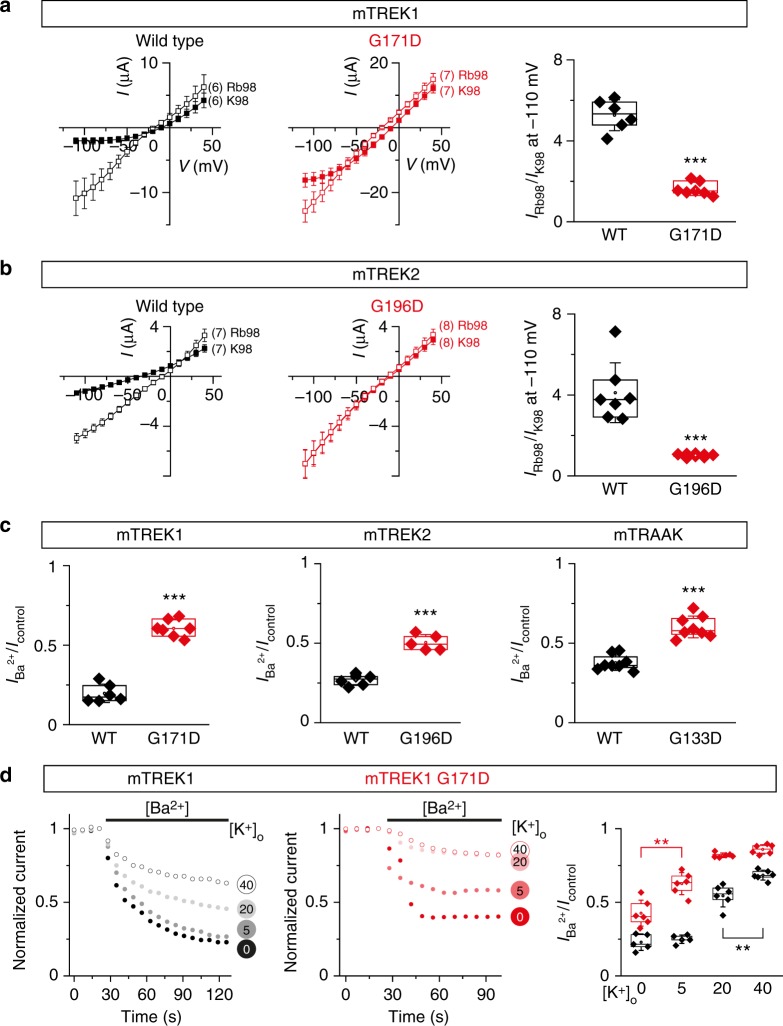
TM2.6 mutations promote gating of mechano-gated K2P channels. **a**, **b** TM2.6 mutation affects TREK1 and TREK2 current increase induced by substituting potassium with rubidium. Current–voltage relationships of mTREK1 and mTREK2 channels; wild-type (black) and TM2.6 mutant (red) at 98 mM extracellular potassium (filled squares) and 98 mM extracellular rubidium (open squares). Each data point represents the mean ± standard error of mean, numbers in parentheses represent the number of oocytes tested for each condition. Rightmost panels: mean relative current increases were 5.3 ± 0.3 and 1.6 ± 0.1 for wild-type and mutant mTREK1, respectively, and 4.1 ± 0.6 and 1.01 ± 0.02 for wild-type and mutant mTREK2, respectively. Currents were recorded 24 h after injection of cRNA at 1 ng/oocyte. **c** Magnitude of Ba^2+^ inhibition is reduced in TM2.6 mutants of mechano-gated K2P channels. Ratios between currents under barium perfusion and currents under control perfusion at 0 mV, were significantly different for wild-type (black) and mutant channels (red). Mean ratios increased from 0.19 ± 0.02 (mTREK1) to 0.6 ± 0.02 (mTREK1 G171D), from 0.26 ± 0.01 (mTREK2) to 0.5 ± 0.01 (mTREK2 G196D), and from 0.37 ± 0.01 (mTRAAK) to 0.6 ± 0.02 (mTRAAK G133D). Currents were recorded 24 h after injection of cRNA at the following concentrations (in ng/oocyte): mTREK1 wild-type, 5; mTREK1 G171D, 1; mTREK2 wild-type, 3; mTREK2 G196D, 1; mTRAAK wild-type, 10; mTRAAK G133D, 0.1. **d** Inhibition of mTREK1 by 6 mM extracellular Ba^2+^ in the presence of increasing concentrations of extracellular K^+^ ([K^+^]_o_). Currents were recorded 24 h after injection of cRNA (mTREK1, black, 5 ng/oocyte; mTREK1 G171D, red, 1 ng/oocyte). Left and middle panels show normalized responses of one representative oocyte to barium challenge in the presence of [K^+^]_o_ (0, 5, 20, and 40 mM). Right panel: ratio between currents under barium perfusion and currents under control perfusion at 0 mV. Each data point represents one oocyte; center lines, medians; open circles, means; box limits, 25th and 75th percentiles; whiskers, standard deviation. Student’s unpaired *t*-test (except (**b**), Mann–Whitney), **p < 0.01, ***p < 0.001

To characterize the effect of TM2.6 mutations on the gating of mechano-sensitive K2P channels, we first measured the relative increase in inward current when extracellular potassium is replaced by rubidium. Indeed, substitution of potassium by rubidium in TREK1 induces the stabilization of the selectivity filter gate, for example making it more difficult to close the channel by reducing the internal pH^20^. We found that the current increase induced by ion substitution was strongly affected in TREK1 and TREK2 mutant channels. While wild-type currents increased 5 and 4.2-fold in TREK1 and TREK2, respectively, we only observed 1.6 and 1.2-fold increases for TREK1 G171D and TREK2 G196D, respectively (Fig. 3a, b), which is consistent with a positive effect of TM2.6 mutations on the selectivity filter gate.

Next, we analyzed the response of TREK1, TREK2, and TRAAK channels to open channel block by divalent barium ions. Ba^2+^ inhibits these channels in a concentration and time-dependent fashion by competing for the S4 potassium binding site in the selectivity filter. In turn, when channel activity is increased by raising extracellular potassium concentrations or pH (inducing C-type gate stabilization), the magnitude of Ba^2+^ inhibition is reduced because occupancy of S4 by potassium is increased^21–23^. Consistent with an increased basal activity, we found that Ba^2+^ block was indeed significantly reduced in TM2.6 mutants of TREK1, TREK2, and TRAAK (Fig. 3c).

We then tested the dependence of Ba^2+^ inhibition to extracellular potassium ([K^+^]_o_) for TREK1. We again observed that TREK1 G171D was less sensitive to Ba^2+^ inhibition, and hence intrinsically more active (Fig. 3d). Ba^2+^ block was less pronounced at 5, 20, and 40 mM [K^+^]_o_ when we compared wild-type to mutant TREK1. And while the magnitude of inhibition further increased between 20 and 40 mM [K^+^]_o_ for wild-type channels, TREK1 G171D inhibition reached a plateau at 20 mM extracellular potassium, likely reflecting maximal opening of the mutant channel at this concentration (Fig. 3d). In addition, while wild-type channels were inhibited in the same fashion at 0 and 5 mM [K^+^]_o_, the level of Ba^2+^ inhibition was significantly different between the two concentrations in TREK1 G171D. Taken together, these results strongly suggest that TM2.6 mutations promote the gating of mechano-gated K2P channels via the selectivity filter gate.

### TM2.6 mutation increases TRAAK/TREK1 single-channel activity

To analyze in more detail how mutations of TM2.6 affect channel activity, we performed single-channel recording experiments for TRAAK and TREK1 channels.

**Fig. 4:**
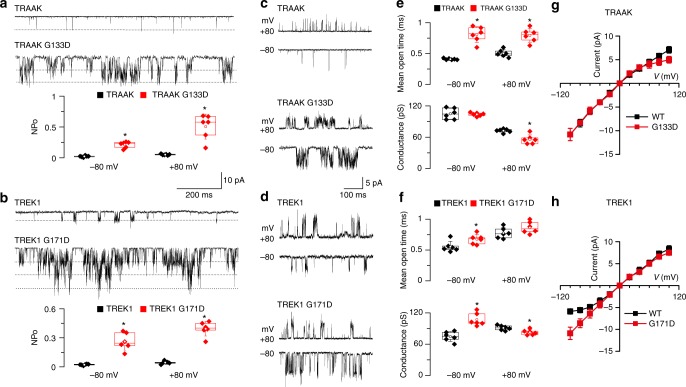
TM2.6 mutations in TRAAK and TREK1 increase single-channel open probability. **a**, **b** Channel opening of TRAAK, TRAAK G133D, TREK1, and TREK1 G171D at −80 mV. Channel activity was determined by NPo analyzed at −80 and +80 mV. **c**, **d** Single-channel openings of TRAAK, TRAAK G133D, TREK1 and TREK1 G171D at two membrane potentials. Currents were recorded in cell-attached patches held at pipette potentials of +80 and −80 mV. Pipette and bath solutions contained 150 mM KCl. **e**, **f** Mean open time for TRAAK, TRAAK G133D, TREK1, and TREK1 G171D. Average unitary conductance for TRAAK, TRAAK G133D, TREK1, and TREK1 G171D. **g**, **h** Current–voltage relationships of single channels. Mean ± standard deviation. Source data are provided as a Source Data file. Each data point represents one recording; center lines, medians; open circles, means; box limits, 25th and 75th percentiles; whiskers, standard deviation. Mann–Whitney, *n* = 6, *p < 0.05

First, we transfected TRAAK, TRAAK G133D, TREK1, and TREK1 G171D into HEK-293 cells and compared channel activity in cell-attached patch recordings. We found that TRAAK G133D and TREK1 G171D activity rose approximately 10-fold compared to wild-type channels in this context. While wild-type TRAAK and TREK1 channel activity was slightly increased at depolarized membrane potentials, TM2.6 mutants were consistently more active for all membrane potentials compared to wild-type (Fig. 4a, b).

Next, we analyzed single-channel properties of wild-type and mutant channels. In both mutants, we could observe flickering behavior and long bursts of channel openings consistent with increased channel activity (Fig. 4c, d). In addition, mean open times were significantly increased compared to wild-type. For example, mean open times of TRAAK channels saw a 2-fold increase from 0.41 ± 0.01 to 0.81 ± 0.13 ms at −80 mV, while TREK1 channel mean open times rose from 0.56 ± 0.09 to 0.68 ± 0.08 ms at −80 mV (Fig. 4e, f).

In contrast, unitary conductance was only modestly affected by TM2.6 mutations in TRAAK and TREK1, and would not explain the dramatic increase in whole-cell current observed in our two-electrode voltage-clamp experiments. We observed differences between wild-type and mutant channels depending on the imposed membrane potential. TRAAK conductance was unchanged by TM2.6 mutation at −80 mV but showed a significant decrease compared to wild-type at +80 mV. TREK1 G171D unitary conductance was significantly higher than wild type at −80 mV, and slightly decreased at +80 mV (Fig. 4e, f). Consistently, the current–voltage relationships of TRAAK and TREK1 (obtained from single-level openings in symmetrical KCl, 150 mM) was also modified. The *I*–*V* relationship of TRAAK G133D showed a slight inward rectification at positive potentials similar to that of TREK2^24^ (Fig. 4g, h). TREK1 produced a clear outward rectification at negative potentials, while TREK1 G171D did not.

Taken together, these results provide direct evidence that introducing an aspartate in TM2.6 of TRAAK and TREK1 dramatically affects single-channel open probability and strongly potentiates the channels’ activity. Our data are consistent with the effect of the TM2.6 G134D mutation reported for Drosophila KCNK0/ORK1^8^, which was found to increase single-channel open probability. This congruence is striking given the low levels of sequence identity between KCNK0 and TRAAK/TREK1 channels (Supplementary Table 1). We therefore propose that increased channel open probability most likely explains the consistent increase in activity of TM2.6 mutants.

### Gradual activation of TREK1 and TASK2

Given the drastic effect of asparagine and aspartate mutations, we wondered whether we could also achieve intermediate levels of channel activation by using different amino acid substitutions at the TM2.6 position. Such allelic series have been reported for TWIK1^25^ and TALK2^7^, but also for members of other ion channel families such as large conductance mechanosensitive channels (MscL) of *Escherichia coli*^26^, voltage-dependent Shaker potassium channels, or the ligand-gated K^+^ channel GsuK^27^.

**Fig. 5:**
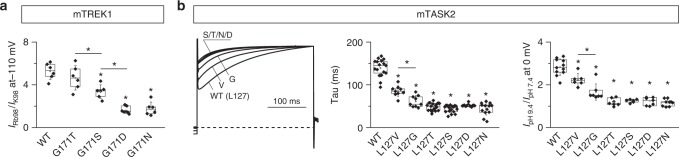
Gradual activation of TREK1 and TASK2 by a series of TM2.6 mutations. **a** Response of different TREK1 TM2.6 mutants to rubidium/potassium ion substitution. Ratios of whole-cell currents recorded at −110 mV for [Rb^+^]_o_ 98 mM and [K^+^]_o_ 98 mM. **b** Gradual increase of TASK2 activation kinetics and response to pH challenge for different TM2.6 mutants. Normalized current traces recorded at +40 mV (left panel) and corresponding activation constants (Tau (ms), middle panel). Ratios of whole-cell current recorded at 0 mV with extracellular pH 9.4 and pH 7.4 (right panel). Currents were recorded 24 h after injection of cRNA at 1 ng/oocyte. Each data point represents one oocyte; center lines, medians; open circles, means; box limits, 25th and 75th percentiles; whiskers, standard deviation. ANOVA, Tukey’s multiple comparison test, *p < 0.05

To test this hypothesis, we generated a series of TM2.6 mutants of the TREK1 and TASK2 channels and compared their biophysical properties with those of wild type channels. First, we used the potassium–rubidium ion substitution assay to compare four TREK1 mutants (G171T, G171S, G171D, G171N). We observed significant differences for S, D, and N mutants compared to wild-type TREK1, and between T and S, or S and D mutants (Fig. 5a). This strongly suggests that these mutants achieve distinct levels of channel function.

Next, we analyzed six different TASK2 mutants (L127V, L127G, L127T, L127S, L127D, L127N) and found gradual effects for activation kinetics and pH-sensitivity. At physiological pH, valine and glycine substitution had intermediate kinetics, while the remaining mutants had comparable, likely maximal, activation kinetics (Fig. 5b, leftmost and middle panel). Using the same mutants, we then compared the stimulating effect of external alkalinization. We found consistent results for the six mutants (Fig. 5b, rightmost panel): L127V and L127G showed intermediate phenotypes, while L127T/S/D/N mutants were indistinguishable.

Finally, we investigated the effect of serine substitution on the single-channel properties of TRAAK and TREK1 channels (Supplementary Fig. 3). Similar to G133D, G133S strongly increased NPo of TRAAK at −80 mV and +80 mV, but did not cause a significant increase in mean open time at +80 mV. For TREK1, while NPo, mean open time, and conductance, were significantly increased by G171D at −80 mV, only mean open time was significantly increased in G171S mutants compared to wild-type.

Taken together these results show that different levels of channel activity can indeed by achieved by engineering different amino acid substitutions in TM2.6, further illustrating the pivotal role played by this residue.

### Gradual activation of the nematode K2P channel TWK-18

Since TM2.6 mutations increase channel gating for all vertebrate channels (Fig. 1b) and for Drosophila KCNK0/ORK1^8^, we wondered whether the function of this residue had been further conserved during evolution. We therefore targeted the diverse family of K2P channels expressed in *C. elegans*.

**Fig. 6:**
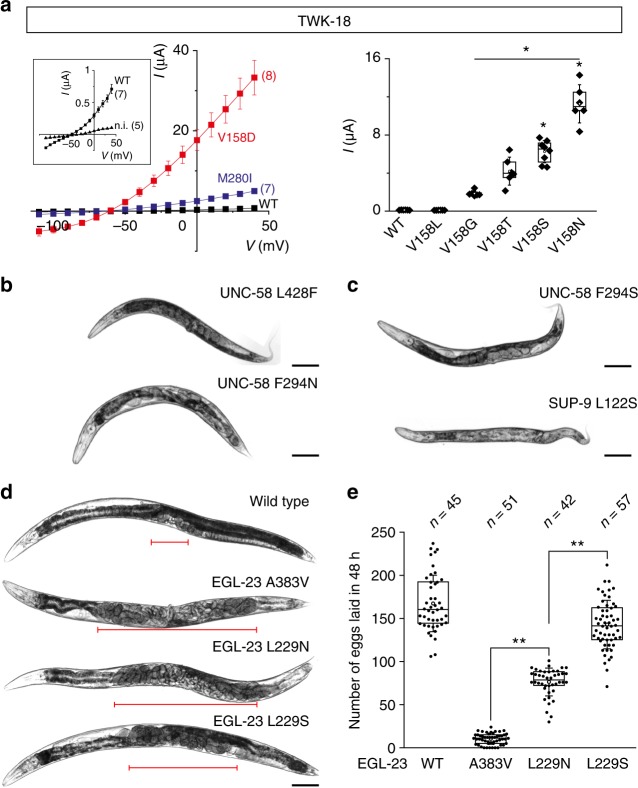
Tuning K2P channel activity in vivo using CRISPR/*Cas9* gene editing in *C*. *elegans*. **a** (left panel) Current–voltage relationships obtained at pH 7.4 in *X*. *laevis* oocytes for TWK-18 wild-type (black squares), TWK-18 M260I (blue squares), and TWK-18 V158D mutant channels (red squares). Inset, current–voltage relationship for wild-type channel and non-injected oocytes at a reduced scale. **a** (right panel) Gradual increase of TWK-18 whole-cell current in a series of TM2.6 mutants. Currents at 0 mV were recorded 24 h after injection of cRNA at 30 ng/oocyte. Each data point represents one oocyte. **b** Representative micrographs of 1-day old adult UNC-58 L428F, UNC-58 F294N, UNC-58 F294S, **c** SUP-9 L122S, and **d** wild type, EGL-23 A383V, EGL-23 L229N, and EGL-23 L229S worms. Red brackets mark the central section of each animal where embryos accumulate. Scale bars, 10 µm. **e** Comparison of egg-laying rates of wild-type worms and *egl-23* gain-of-function alleles. Each data point represents the number of embryos laid by a single animal over a 48-h period. *n*, number of animals tested. Center lines, medians; open circles, means; box limits, 25th and 75th percentiles; whiskers, standard deviation. Kruskal–Wallis, Dunn’s multiple comparison test, *p < 0.01

To date, only the TWK-18 channel has been functionally characterized using heterologous expression systems^5^. TWK-18 is a potassium-selective outwardly-rectifying channel with a steep temperature-dependence between 25 and 35 °C. To test whether TM2.6 mutation could also increase TWK-18 activity, we compared the TM2.6 mutant TWK-18 V158D with wild-type TWK-18 and the previously described gain-of-function mutant TWK-18 M280I (*cn110*). Consistent with our previous results, we found that TWK-18 V158D was significantly more active than wild-type and M280I mutants at 22 °C when expressed in *Xenopus* oocytes (Fig. 6a, left panel). We then generated additional TM2.6 amino acid substitutions in TWK-18 to test whether we could also achieve gradual activation. Indeed, we found that different residues lead to different levels of whole-cell current, consistent with progressive activation of the TWK-18 channel (Fig. 6a, right panel).

### Rational design of gain-of-function mutants in *C*. *elegans*

Despite the fundamental role of K2P channels in cellular physiology^1,28,29^, some basic questions about their biology are still largely unexplored. In particular, comparatively little is known about the precise molecular and cellular processes that determine the number of active channels and their distribution at the cell surface in vivo. Unbiased forward genetic screens in model organisms are well suited to dissect such complex cellular processes and gene regulatory networks in vivo, and gain-of-function mutants of potassium channels are ideal starting points for such screens. For example, genetic modifier screens targeting the SUP-9 channel have identified a diverse set of proteins that are required for trafficking and function of this TASK-related K2P channel^6,30,31^.

We hypothesized that TM2.6 mutation could be a powerful strategy to rationally design hyperactive K2P channel mutants that would open the way for genetic screens. As a proof of principle, we used CRISPR/*Cas9*-based gene editing to target four channels (*twk-18*, *unc-58*, *egl-23*, and *sup-9*) for which gain-of-function mutants with severe and easily observable phenotypes had been reported in the literature.

*twk-18* is exclusively expressed in *C*. *elegans* body wall muscles^32^. Hyperactivation of TWK-18 by two different point mutations (*cn110/*M280I and *e1913*/G165D) leads to flaccid paralysis that can be rapidly induced and reverted in temperature-shift experiments^5^. *unc-58(e665)* (L428F) mutants are short and essentially unable to move forward or backward on solid media^33^. They display a straight body posture accompanied by a rapid rotation around their anteroposterior axis. *egl-23(n601)* (A383V) mutants are unable to lay embryos likely due to the hyperpolarization of vulval muscles (SEM and TB, unpublished). Finally, gain-of-function mutants in which SUP-9 is hyperactive display a characteristic rubberband phenotype in which worms first contract and then rapidly relax their body when prodded on the head^6^.

We engineered asparagine mutations in TM2.6 for *twk-18*, *unc-58*, *egl-23*, and *sup-9*. Except for *sup-9*, mutant worms harboring the desired point mutations could be readily identified in the F1 progeny of injected worms owing to the dominant effect of these mutations (Fig. 6b, d). First, we found that TWK-18 V158N mutant worms showed the same flaccid paralysis as canonical M280I gain-of-function mutants. Second, UNC-58 F294N mutants were severely paralyzed and short but differed qualitatively from the canonical UNC-58 L428F mutant. Indeed, they did not produce the characteristic shaking behavior, and had increased body curvature on agar plates. Third, egg-laying was strongly impaired in EGL-23 L229N mutants (Fig. 6d, e). As expected, fertilized embryos quickly accumulated within the parent, eventually leading to the formation of a bag-of-worms once these animals had completed their embryonic development. Finally, despite multiple attempts, we failed to obtain the SUP-9 L122N mutant. This may be explained by the fact that even moderate gain-of-function mutations of *sup-9* can only be maintained as heterozygous strains^6^, and homozygous animals fail to develop during embryogenesis.

### Allelic series produce gradual phenotypes in *C*. *elegans*

In heterologous expression systems such as *Xenopus* oocytes, it is possible to control the level of ion channel expression by titrating the amount of injected cRNA. This is no longer possible when mutations are introduced into the genome by gene editing. As we found for SUP-9, asparagine mutations in TM2.6 may sometimes interfere with normal development or limit viability. We thus wondered whether we could generate weak or intermediate gain-of-function mutants in vivo by using different amino acid substitutions.

We again used CRISPR/*Cas9* gene editing to introduce different TM2.6 mutations in *twk-18*, *unc-58*, *egl-23*, and *sup-9*. In each case, we could identify CRISPR/*Cas9*-edited animals based on the characteristic locomotor (*twk-18*, *unc-58*), egg-laying (*egl-23*) or developmental phenotypes (*sup-9*). First, we were able to generate homozygous SUP-9 L122S mutants, contrary to our previous attempts with the L122N mutation. These worms however grew very slowly and had strong morphological defects (Fig. 6c). Since they were nevertheless viable, we concluded that SUP-9 L122S is likely a weaker gain-of-function allele than L122N and that channel activity can indeed be set to different levels using serine vs. asparagine substitutions.

Next, we built serine and threonine mutants for *twk-18*, *unc-58*, and *egl-23* to test whether we could detect gradual channel activation using behavioral variation as a proxy. In the case of the temperature-sensitive TWK-18 channel, we could define a clear allelic series by comparing the level of paralysis of *twk-18* mutants at different temperatures. At extreme temperatures, all alleles behaved similarly, i.e., they remained mobile at 15 °C and were paralyzed at 25 and 30 °C. We could however observe clear differences at 20 °C when we measured the proportion of animals that were able to crawl out of a 1 cm-wide circle within a two-and-a-half-hour timespan. At 20 °C, 95% of M280I (*n* = 40), 73% of V158S (*n* = 40), and 63% of V158T (*n* = 40) had crossed this limit. In contrast, only 22% of the V158N mutants (*n* = 40) had moved significantly over this time period. Using a similar behavioral test for UNC-58, we could also observe progressive phenotypes. 87% of F294S (*n* = 45) and 49% of F294N (*n* = 45) had crossed the perimeter of the circle after an 8 h, compared to 9% of L428F (*n* = 45) mutants.

In the case of *egl-23*, we monitored the rate of egg-laying in the first 48 h after the L4 to adult molt (Fig. 6e). We compared the canonical *n601* (A383V) mutant, and the TM2.6 mutants L229N and L229S. The A383V mutation had the strongest effect on the egg-laying rate. L229N resulted in slightly higher egg-laying rates (i.e., a less pronounced egg-laying defect), while L229S mutants were able to lay eggs at a rate slightly lower than wild-type. This gradual effect was also clearly visible in 1-day old adult animals (Fig. 6d, e). Indeed, wild-type worms carry only a limited number of fertilized eggs at this time point since they lay embryos soon after fertilization. In contrast, A383V and L229N mutants rapidly accumulate fertilized embryos which eventually leads to death by matricide after 48 h. L229S mutants had an intermediate phenotype consistent with their ability to lay eggs and increased survival time. Eventually, all mutant worms however died due to the accumulation and hatching of their progeny within the parent.

These experiments further validate the notion that TM2.6 is a crucial residue that controls the gating of most, if not all K2P channels. They also demonstrate that channels can be activated progressively in vivo using various TM2.6 substitutions, highlighting the versatility of our strategy.

## Discussion

We report here that a single conserved residue in the second transmembrane segment of vertebrate and invertebrate two-pore domain potassium channels plays a key role in the control of K2P channel gating. Mutating this residue (TM2.6) results in higher channel activity in heterologous expression systems and in vivo in *C. elegans*. Higher channel activity results from a dramatic increase of channel open probability, and not from a change in surface expression nor unitary conductance. Furthermore, it is possible to engineer gain-of-function mutants with different levels of activity by replacing TM2.6 with a series of amino acids, progressively tuning the activity of K2P channels. TM2.6 systematically increases channel activity in most if not all K2Ps. This universality is remarkable given the low sequence conservation between the vertebrate and invertebrate channels we have tested and raises the question of the mechanism of action of these mutations.

Available crystal structures position TM2.6 on the intracellular side of the second transmembrane domain, exposed to a large cytoplasmic vestibule, and in close proximity to the intracellular part of the selectivity filter^16–18,34^. This location suggests that TM2.6 residues could affect three previously-described gating mechanisms.

Contrary to other potassium channels, K2P channels do not possess a clear bundle-crossing gate or inner gate. Their principal gate is therefore situated at or close to the selectivity filter, also known as a C-type gate because it resembles the C-type inactivation gate of voltage-gated potassium channels^4,18,20,35–38^. In most K2Ps, this C-type gate is regulated by variations in pH and is stabilized by increasing extracellular potassium concentrations^39,40^. Mutations and small molecules have been shown to stabilize this filter gate in different K2P channels^4,18^. Our data are consistent with an effect of TM2.6 mutations on the selectivity filter gate.

Interestingly, a novel type of inner gating mechanism has been proposed recently for TWIK1. Molecular dynamics simulations tracking the movements of water molecules within the intracellular vestibule of the channel found that stochastic de-wetting close to the TM2.6 leucine residue creates a hydrophobic barrier opposing efficient ion passage through the conduction pathway^25,41^. Disrupting this hydrophobic barrier by replacing the leucine with a polar or negatively-charged residue maintained water occupancy within the inner pore and dramatically increased whole-cell currents^25^. Similar hydrophobic barriers have emerged from the study of model nanopores and have been shown to limit ion conduction in ligand-gated neurotransmitter receptors and ion channels^41,42^. For example, mutations disrupting hydrophobic barriers affect current flow through two prokaryotic potassium channels (GsuK, ligand-gated K^+^ channel from *Geobacter sulfurreducens*; MthK, Ca^2+^-gated K^+^ channel from *Methanobacterium thermoautotrophicum*) and increase open probabilities^29,43^.

A third gating mechanism has been proposed for mechano-sensitive channels of the TREK/TRAAK family in which lateral fenestrations close to the selectivity filter allow the passage of acyl chains belonging to fatty acids of the upper membrane leaflet. Access of lipids to the conduction pathway relies on conformational changes of TM4^34,44,45^. In K2Ps, TM2 of one subunit is opposed to TM4 of the second subunit thereby creating two fenestrations possibly accessible by lipids. Given the central position of TM2.6 within the second transmembrane domain, amino acid substitutions (either charged, hydrophilic, and/or bulky) of TM2.6 could also alter this proposed lipid gating mechanism.

We have found that the majority of K2P channels possess hydrophobic residues at the TM2.6 position (Fig. 1a, Supplementary Fig. 2). Therefore, disruption of a hydrophobic gate is an attractive model that would explain how K2P channels with very different overall primary sequences could all be similarly activated. Furthermore, simulations of model nanopores have demonstrated that hydrophobic barriers can be disrupted by modulating the hydrophobicity of the pore^42^. Consistent with this idea, we found that different hydrophilic or hydrophobic residues produced different levels of channel activity, both in vitro and in vivo. A few K2P channels do not have hydrophobic TM2.6 residues. Notably, a glycine residue is found in Drosophila KCNK0/ORK1 and vertebrate TREK1, TREK2, and TRAAK channels. Molecular dynamics simulations of TREK2 have failed to detect significant de-wetting^46^. Rather different lines of evidence suggest that mutating this glycine residue to an aspartate increases channel gating via the selectivity filter gate^8^.

In conclusion, the exact mechanisms by which TM2.6 mutations promote channel activation remain to be fully elucidated and may vary depending on the channel. Furthermore, it may be difficult to dissociate hydrophobic and C-type gating since TM2.6 is close to the selectivity filter. Depending on the channel, TM2.6 mutation may thus differentially affect the selectivity filter gate, the inner hydrophobic gate, and gating by membrane lipids.

Given the striking phenotypes of the TM2.6 mutants in *C*. *elegans*, it could appear surprising that they had not been identified in forward genetic screens. A likely explanation is that two or even three base changes are required to introduce these amino acid substitutions. Concomitant mutation of two DNA bases is highly unlikely when using chemical mutagens such as EMS or ENU. Consistently, preliminary experiments targeting other *C*. *elegans* K2P channels have yielded additional mutants with very strong behavioral defects akin to the phenotypes of classical *uncoordinated* mutants (unpublished). Such gain-of-function mutants will be ideal starting points for forward genetic screens aiming to identify genes that are required for channel expression, trafficking and functional modulation in vivo.

In the future, TM2.6 mutations could also be used to identify pharmacological compounds targeting K2P channels. In particular, yeast-based screening strategies have proven to be successful for the discovery of new ion channel modulators^47^ and for the identification of specific blocker sites^48^. By using TM2.6 mutants described here, this approach can now be envisioned for K2P channels beyond the highly active TREK1 channel (keeping in mind that TM2.6 mutations may not always fully mirror the pharmacology of wild-type channels). Indeed, specific compounds targeting K2P channels are of great interest, since K2Ps represent important targets for neurological, cardiovascular, and endocrine disorders. For example, gain-of-function mutations in human TALK2 have been shown to cause severe cardiac conduction disorder^7^. A polymorphism in the pancreatic channel TALK1 causes a reduction in β-cell excitability and glucose-stimulated insulin secretion, which could explain increased type 2 diabetes susceptibility^49^. And a mutation increasing TRAAK activity has been linked to a novel neurodevelopmental syndrome known as FHEIG, for facial dysmorphism, hypertrichosis, epilepsy, intellectual disability/developmental delay, and gingival overgrowth^50^.

Presently, there are no indications about the possible effects of increased channel activity for other human K2P channels. By introducing TM2.6 mutations using CRISPR/*Cas9*-based gene editing, it will be possible to create well-defined cellular systems or model organisms that could help to predict the functional consequences of increased activity for any K2P channel, while circumventing the pitfalls of standard overexpression strategies. In turn, these model systems could then be used to identify and validate pharmacological agents that modulate the activity of K2P channels.

## Methods

### Expression plasmids

cDNA sequences were amplified using Phusion high-fidelity DNA polymerase (ThermoFisher Scientific) and assembled into the *Xenopus* oocyte expression vector pTB207^51^ by 2- or 3-fragment Isothermal Ligation using the primer combinations indicated in Supplementary Table 5. PCR-amplified sequences were validated by Sanger sequencing.

The resulting vectors used in this study are:

pOA23 hTASK1, pOA24 hTASK1 L122N, pOA21 hTASK3, pOA22 hTASK3 L122N, pOA25 mTASK2 WT, pNZ20 mTASK2 L127N, pIBS25 mTASK2 L127D, pIBS26 mTASK2 L127S, pIBS27 mTASK2 L127T, pIBS28 mTASK2 L127V, pIBS29 mTASK2 L127G, pIBS18 mTREK1 WT, pIBS19 mTREK1 G171D, pIBS20 mTREK1 G171N, pIBS21 mTREK1 G171S, pIBS22 mTREK1 G171T, pIBS23 mTREK1 G171L, pIBS24 mTREK1 G171V, pOA26 mTREK2, pOA27 mTREK2 G196D, pOA28 mTRAAK, pOA29 mTRAAK G133D, pOA30 mTRESK, pOA31 mTRESK F156N, pTB301 TWK-18, pTB314 TWK-18 M280I, pNZ19 TWK-18 V158D, pIBS30 TWK-18 V158N, pIBS31 TWK-18 V158S, pIBS32 TWK-18 V158T, pIBS33 TWK-18 V158L, pIBS34 TWK-18 V158G. hTWIK1, rTWIK2, hTALK1, hTALK2 and hTHIK2 are inserted into the pLIN oocyte expression vector. Additional mutations were introduced by PCR using Pfu Turbo DNA polymerase (Agilent Technologies). hTWIK1-AA was previously described in ref. ^9^, hTHIK2-5RA in ref. ^10^, and rTWIK2-LY in ref. ^52^. For mammalian cell electrophysiology, K2P channel cDNA sequences were inserted into the pIRES2-EGFP vector. For FACS analysis, hTWIK1 AA, mTASK3, and mTREK1 were tagged with HA and GFP, and cloned into a pcDNA3 vector. One or two copies of the hemagglutinin (HA) epitope (YPYDVPDYA) were inserted into the extracellular domain connecting the second pore helix (Ph2) and TM4 transmembrane segment. The GFP coding sequence was added at the cytoplasmic C-terminus of each channel.

### Oocyte electrophysiology

Capped RNAs were synthesized in vitro from linearized expression vectors using the T7 mMessage mMachine kit (Ambion, Austin, TX, USA).

Defolliculated *X*. *laevis* oocytes (Ecocyte Bioscience, Dortmund, Germany) were injected with 50 nL containing between 50 pg and 50 ng of cRNA depending on the K2P expression rate (concentrations are indicated in Supplementary Table 1). Oocytes were kept at 18 °C in ORII Calcium solution containing (in millimolar): 82.5 NaCl, 2 KCl, 1 MgCl_2_, 0.7 CaCl_2_, 5 HEPES, gentamicin (25 µg/mL), pH 7.5 (with TRIZMA-Base).

Two-electrode voltage-clamp (TEVC) experiments were performed 24–72 h after the microinjection. Oocytes were mounted in a small home-made recording chamber and continuously superfused with the ND96 standard solution containing (in millimolar): 96 NaCl, 5 KCl, 1.8 CaCl_2_, 2 MgCl_2_, 5 HEPES. pH 7.4 was adjusted with Trizma base. High potassium and high rubidium external solutions (in millimolar): 98 KCl or 98 RbCl, 1.8 CaCl_2_, 2 MgCl_2_, 5 HEPES. pH 7.4 was adjusted with Trizma base. For pH challenge experiments, Trizma base was used to buffer the solution for pH values over 8.0. Barium chloride (Sigma-Aldrich) was prepared as 1 M stock solution in H_2_O and used at 6 mM final concentration in ND96 solution.

Macroscopic currents were recorded using a Warner Instrument OC-725 amplifier, filtered at 10 kHz, digitized using a Digidata-1322 (Axon Instrument). For current visualization and stimulation protocol application, we used Axon pClamp 9 software (Molecular Devices, Sunnyvale, CA). Recording electrodes were pulled to 0.2–1.0 MΩ by using a horizontal puller (Sutter Instrument, Model P-97, USA) and filled with 3 M KCl. Currents were recorded in response to two recording protocols. The first was a voltage-step protocol consisting of a pre-pulse of −80 mV (80 ms duration) from a holding potential of −60 mV, followed by steps (300 ms duration) from −110 to 40 mV, and return to a −60 mV holding potential. Current–voltage curves were obtained by plotting the steady-state currents at the end of each voltage step. The second protocol was a ramp protocol used for time course manipulations, consisting on a pre-pulse of −110 mV (100 ms duration), from a holding potential of −60 mV, followed by a ramp from −110 to 40 mV over 3.5 s. This protocol was automatically repeated every 7 s. The time course of the activation for mTASK-2 channels (*τ*, ms) was calculated by fitting the current trace obtained at 0 mV with a single exponential suite of pClamp 9.

### Flow cytometry

HEK-293 cells were grown in 60 mm dishes and transiently transfected with HA/GFP-tagged K2P vectors using JetPEI (Polyplus transfection). 24 h after transfection, cells were gently harvested by using PBS, 10 mM EDTA, then centrifuged for 5 min at 350 g and resuspended in PBS, 5% horse serum incubation solution at 5 × 10^6^ cells per mL. 100 μL of the cells were then incubated for 2 h on ice with anti-HA antibody (Sigma-Aldrich, HA-7 clone, 1/1000), followed by 1 h with Alexa594-conjugated anti-mouse antibody (Molecular Probes, 1/1000). Cells were washed once with incubation solution and resuspended in PBS. Quantification was performed on a BD LSR2 Fortessa using 525/50 bandpass (excitation GFP 488 nm), 605/40 bandpass (excitation Alexa594 561 nm) and 450/50 (excitation Dapi 405 nm) filter sets. Live single cells were identified as DAPI-negative and based on forward scatter/side scatter profiles. The GFP-positive gate (GFP+) and red-positive gate (Alexa594+ or HA+) were set manually using the following control conditions: (c1) the background level of red fluorescence was determined by measuring fluorescence intensity of empty vector transfected cells incubated with antibodies (c2) the GFP-positive window was estimated from unlabeled transfected cells and (c3) the HA-positive window with cells transfected with an HA-tagged construct incubated with antibodies. Cytometry data were analyzed using the BD CellQuest Pro software (BD Biosciences).

### Single-channel analysis

HEK-293 cells (obtained from the Korean Cell Line Bank) were seeded at a density of 2 × 10^5^ cells per 35 mm dish 24 h prior to transfection in Dulbecco’s modified Eagle’s medium (DMEM) containing 10% FBS. HEK-293 cells were transfected with mTREK1, mTREK1 G171S, mTREK1 G171D, mTRAAK, mTRAAK G133S, or mTRAAK G133D in pIRES-eGFP DNA using LipofectAMINE2000 and OPTI-MEM I Reduced Serum Medium (Life technologies, Grand Island, NY, USA). Cells expressing green fluorescence were detected with the aid of a Nikon microscope equipped with a mercury lamp light source. Cells were used 1–3 days after transfection.

All recordings were performed using a patch clamp amplifier (Axopatch 200, Axon Instruments, Union City, CA, USA). Single-channel currents were filtered at 2 kHz using an 8-pole Bessel filter (−3 dB; Frequency Devices, Haverhill, MA) and transferred to a computer using the Digidata 1320 interface (Axon) at a sampling rate of 20 kHz. Threshold detection of channel openings was set at 50%. Single-channel currents were analyzed with the pCLAMP program (version 10, Axon). The filter dead time was 100 μs (0.3/cutoff frequency) for single-channel analysis, therefore, events lasting less than 50 μs were not detected. Channel activity (NP_o_, where N is the number of channels in the patch and P_o_ is the probability of a channel being open) was determined from ~1–2 min of current recording. In experiments using cell-attached patches, the pipette and bath solutions contained (mM): 150 KCl, 1 MgCl_2_, 5 EGTA, and 10 HEPES (pH 7.3). All experiments were performed at ~25 °C.

### *C*. *elegans* experiments

All strains described in this study were built using N2 (obtained from the CGC) as a wild-type starting strain. Strains were fed OP50 and grown at 20 °C unless otherwise noted. Strains generated for this study are listed in Supplementary Table 2.

All TM2.6 mutants were generated using CRISPR/*Cas9*-based homologous recombination by injecting *Cas9* ribonucleoprotein complexes into the syncytial gonad of 1-day old N2 hermaphrodites alongside single-strand DNA oligonucleotide repair templates. First, guide RNA duplexes were formed in vitro by incubating 3 µL of crRNA with 3 µL of tracrRNA (100 µM stock solution each, in Nuclease-Free Water, IDT) in Nuclease-Free Duplex Buffer (IDT) in a final volume of 10 µL. RNA duplexes were then incubated at 95 °C for 5 min, followed by 5 min incubation at room temperature. Next, 1.5 µL of crRNA:tracrRNA duplex was added to 15 µg of recombinant *Cas9* protein (Alt-R *S*. *p*. *Cas9* Nuclease 3NLS, IDT), followed by 125 pmol of single-strand DNA repair template and nuclease-free water up to a final volume of 10 µL. This mix was injected into adult hermaphrodites after centrifugation (10 min, 10,000*g*, 4 °C). Injected worms were raised at 25 °C for 2–3 days, after which mutant worms were identified in the F1 progeny based on expected phenotypes of known gain-of-function mutants. CRISPR/*Cas9-*induced molecular lesions were confirmed by Sanger sequencing for all alleles generated in this study. Genome engineering reagents are listed in Supplementary Table 3 and 4.

### Microscopy

*C*. *elegans* images were acquired using brightfield illumination on a Zeiss Z1 AxioImager at ×5 magnification. Worms were immobilized using M9 buffer supplemented with 50 mM sodium azide.

### Reporting summary

Further information on experimental design is available in the Nature Research Reporting Summary linked to this article.

## Supplementary information

Supplementary Information

Reporting Summary

Source Data

## Data Availability

Data supporting the findings of this manuscript are available from the corresponding authors upon reasonable request. A reporting summary for this article is available as a Supplementary Information file. The source data underlying Figs. 1c, 2a, b, 3a–d, 4a, b, e, f, 5a, b, 6a, e, and Supplementary Fig. 3 are provided as a Source Data file.
