# Supplementary material for: Mutation of a single residue promotes gating of vertebrate and invertebrate two-pore domain potassium channels
Source: Nat Commun. 2019 Feb 15;10:787. doi: 10.1038/s41467-019-08710-3 (PMC6377628; doi:10.1038/s41467-019-08710-3)
Supplement: Supplementary file 1 — Supplementary Information [file 41467_2019_8710_MOESM1_ESM.pdf]

Supplementary Information

Mutation of a single residue promotes gating of vertebrate and invertebrate two-pore domain potassium channels

Ben Soussia*, El Mouridi*, *et al.*

Nature Communications (2019)

Supplementary Figure 1 – One-to-one sequence identity matrix based on clustal omega alignement. Genbank identifiers for aligned sequences are listed below. N- and C-terminal cytoplasmic portions were truncated before alignment.

	TWIK1	TWIK2	TRAAK	TREK1	TREK2	TASK1	TASK3	TASK5	TASK2	TALK1	TALK2	TRESK	THIK1	THIK2	KCNK7	
TWIK2	47															
TRAAK	33	35														
TREK1	31	31	52													
TREK2	33	29	56	70												
TASK1	32	29	31	33	35											
TASK3	32	29	31	34	32	74										
TASK5	31	29	31	35	33	67	68									
TASK2	29	30	33	32	33	33	32	34								
TALK1	34	33	39	38	38	37	35	38	39							
TALK2	27	31	38	34	32	30	30	33	37	44						
TRESK	26	25	25	28	30	31	32	29	21	29	23					
THIK1	32	25	28	29	28	32	31	29	26	30	24	26				
THIK2	32	31	27	30	27	30	28	30	26	29	23	25	70			
KCNK7	42	36	29	27	28	26	26	29	24	26	24	21	21	25		
EGL-23	23	24	28	26	26	29	29	28	26	25	24	25	20	23	21	EGL-23
UNC-58	23	25	25	22	23	24	23	24	24	27	23	27	20	20	19	UNC-58
SUP-9	27	25	31	33	32	58	58	52	31	33	30	30	31	31	25	SUP-9
TWK-1	29	27	24	25	25	27	26	24	23	24	21	26	21	19	26	TWK-1
TWK-2	25	26	25	25	25	27	28	26	28	26	22	27	22	22	22	TWK-2
TWK-3	24	25	20	22	20	23	21	24	21	20	20	28	21	22	21	TWK-3
TWK-4	29	29	26	27	29	27	28	26	27	30	26	28	25	25	25	TWK-4
TWK-5	21	22	25	25	22	23	24	23	23	23	28	23	20	18	13	TWK-5
TWK-6	22	20	24	21	21	20	21	20	18	26	22	24	21	21	18	TWK-6
TWK-7	28	28	30	26	29	30	29	30	29	31	28	28	23	23	20	TWK-7
TWK-8	24	23	27	25	24	26	28	25	27	29	27	25	25	23	21	TWK-8
TWK-9	27	25	29	25	27	29	31	29	30	27	24	24	22	24	22	TWK-9
TWK-10	23	20	24	25	23	22	22	23	27	23	23	26	20	22	17	TWK-10
TWK-11	28	24	26	27	25	28	28	28	25	30	23	26	20	21	26	TWK-11
TWK-12	21	21	25	23	27	25	22	24	22	26	25	21	20	18	16	TWK-12
TWK-13	27	24	32	29	28	32	30	32	26	29	26	25	25	22	23	TWK-13
TWK-14	27	24	22	26	23	28	26	25	25	25	24	23	39	39	18	TWK-14
TWK-16	20	20	26	24	24	21	23	25	24	24	22	19	20	21	24	TWK-16
TWK-17	22	24	25	25	25	28	29	27	27	25	23	22	23	22	20	TWK-17
TWK-18	25	26	24	27	27	27	26	26	23	29	27	30	22	23	20	TWK-18
TWK-20	27	27	28	30	28	47	47	43	30	32	28	27	30	29	24	TWK-20
TWK-21	27	22	30	29	27	26	27	27	28	29	29	26	24	25	27	TWK-21
TWK-22	26	23	26	26	24	25	25	25	26	27	28	26	22	25	23	TWK-22
TWK-23	25	21	27	29	29	29	27	27	25	27	23	27	24	23	27	TWK-23
TWK-24	21	21	22	23	25	27	28	23	20	21	23	28	20	17	20	TWK-24
TWK-25	26	26	23	24	21	23	23	23	23	28	26	25	22	21	22	TWK-25
TWK-26	25	25	26	25	23	24	23	24	23	26	24	25	20	22	22	TWK-26
TWK-28	24	26	27	27	26	30	29	31	29	28	29	28	20	24	22	TWK-28
TWK-29	22	20	22	22	22	24	24	23	23	24	17	25	17	16	19	TWK-29
TWK-30	25	24	24	24	25	26	25	24	27	25	23	23	25	24	22	TWK-30
TWK-31	26	25	26	27	28	25	27	26	25	27	24	28	24	24	22	TWK-31
TWK-32	24	21	23	21	23	21	21	22	22	23	20	23	17	19	20	TWK-32
TWK-33	27	22	20	22	20	23	22	25	22	25	25	25	21	20	21	TWK-33
TWK-34	28	22	20	23	21	22	22	25	22	26	26	22	21	19	20	TWK-34
TWK-35	30	25	27	25	28	28	28	28	26	27	26	28	22	24	25	TWK-35
TWK-36	24	24	24	25	23	26	24	26	23	24	26	25	20	18	21	TWK-36
TWK-37	24	25	21	24	24	28	29	25	24	23	24	23	21	23	17	TWK-37
TWK-39	30	27	27	28	27	29	30	32	24	26	27	26	26	28	22	TWK-39
TWK-40	25	26	27	27	31	27	26	25	28	29	27	28	23	26	23	TWK-40
TWK-42	22	22	26	24	28	27	27	26	23	25	25	21	23	21	20	TWK-42
TWK-43	24	24	25	26	26	26	26	27	25	25	22	23	20	22	22	TWK-43
TWK-44	25	25	24	23	24	24	26	24	23	25	25	26	20	22	21	TWK-44
TWK-45	26	26	24	22	22	22	22	23	22	24	22	24	20	20	23	TWK-45
TWK-46	37	35	29	32	31	29	32	28	28	29	25	27	25	24	24	TWK-46
TWK-47	20	17	18	19	18	20	19	22	20	20	21	20	17	16	16	TWK-47
TWK-48	27	25	27	28	29	29	31	30	24	29	24	30	25	25	21	TWK-48
ORK1/KCNK0	28	24	29	28	26	26	25	24	30	29	26	22	23	25	19	ORK1/KCNK0
dTask-6	28	25	28	31	32	55	57	52	31	33	28	29	33	31	24	dTask-6
dTask-7	28	25	30	29	31	54	55	49	29	34	29	27	32	30	22	dTask-7
Sandman	27	25	25	28	27	24	26	25	25	25	27	25	23	23	22	Sandman
CG10864	22	24	26	27	26	22	24	26	23	23	23	27	25	24	22	CG10864
CG34396	28	24	27	27	24	30	30	30	27	26	27	25	25	24	25	CG34396
CG43155	31	29	28	28	29	30	30	31	31	31	31	28	25	25	24	CG43155
Galene	28	28	31	31	30	34	34	34	25	28	30	27	27	27	22	Galene
	TWIK1	TWIK2	TRAAK	TREK1	TREK2	TASK1	TASK3	TASK5	TASK2	TALK1	TALK2	TRESK	THIK1	THIK2	KCNK7	

Supplementary Figure 1 - continued.

	EGL-23	UNC-58	SUP-9	TWK-1	TWK-2	TWK-3	TWK-4	TWK-5	TWK-6	TWK-7	TWK-8	TWK-9	TWK-10	TWK-11	TWK-12	TWK-13	TWK-14	TWK-16	TWK-17	TWK-18
UNC-58	22																			
SUP-9	29	26																		
TWK-1	26	23	26																	
TWK-2	28	26	29	23																
TWK-3	26	22	24	24	29															
TWK-4	30	25	26	25	30	32														
TWK-5	24	19	23	25	24	24	21													
TWK-6	18	18	22	21	20	23	20	21												
TWK-7	29	27	26	24	41	31	34	25	21											
TWK-8	28	26	26	22	29	22	25	23	24	26										
TWK-9	42	23	27	23	26	21	27	24	20	28	22									
TWK-10	23	20	22	24	28	37	28	24	20	29	22	26								
TWK-11	27	25	26	24	29	23	27	18	20	29	37	26	27							
TWK-12	23	23	26	18	22	21	25	18	21	25	27	22	19	28						
TWK-13	27	28	29	25	33	28	31	27	25	37	25	28	28	26	21					
TWK-14	20	17	28	24	23	18	20	23	22	19	22	23	19	22	20	22				
TWK-16	29	19	23	22	22	22	24	22	17	23	22	31	21	26	15	27	16			
TWK-17	26	38	27	23	27	24	26	18	19	28	23	26	22	25	23	24	19	24		
TWK-18	24	24	26	31	27	23	28	22	25	29	29	25	20	29	28	27	28	25	21	
TWK-20	28	24	52	21	26	21	28	22	20	25	27	26	25	24	22	27	24	22	25	24
TWK-21	27	21	29	25	24	21	27	24	23	24	28	26	22	30	23	26	25	25	21	30
TWK-22	23	22	27	23	26	23	26	23	23	25	30	24	22	31	26	26	23	24	20	28
TWK-23	33	27	29	25	30	25	30	27	21	27	27	32	24	30	20	33	22	25	25	26
TWK-24	21	23	29	23	23	22	22	20	20	20	26	23	19	31	26	24	21	22	22	26
TWK-25	22	22	22	20	23	25	23	18	21	27	22	26	22	22	18	25	17	20	23	24
TWK-26	22	22	26	23	26	25	23	21	21	26	24	24	25	28	22	26	21	23	26	29
TWK-28	29	31	29	25	37	28	31	28	22	41	26	26	31	30	23	37	26	24	28	26
TWK-29	26	21	22	25	24	20	22	24	18	22	22	24	22	24	17	27	19	37	23	25
TWK-30	30	25	24	23	28	28	31	26	16	31	23	28	27	25	18	32	24	20	25	25
TWK-31	24	29	26	23	30	25	26	24	24	28	34	22	24	31	28	29	24	28	24	34
TWK-32	25	25	23	23	33	26	32	24	18	31	21	20	26	22	20	31	21	21	19	20
TWK-33	24	20	22	18	27	24	22	18	23	26	22	26	26	23	19	27	16	22	22	28
TWK-34	24	21	22	18	27	23	23	20	24	27	23	26	24	22	20	26	17	20	23	27
TWK-35	26	26	33	21	27	28	25	25	22	25	31	25	24	33	26	27	22	23	24	28
TWK-36	24	19	26	19	30	23	23	18	20	27	24	26	23	26	20	24	16	22	23	28
TWK-37	28	20	24	23	23	23	25	25	18	21	22	25	20	24	24	22	23	20	23	30
TWK-39	23	22	31	25	28	26	24	23	22	29	24	24	26	24	22	25	25	26	24	25
TWK-40	30	29	32	27	37	27	34	24	22	41	24	27	27	28	27	35	23	24	27	27
TWK-42	24	23	30	20	26	22	26	18	23	25	28	23	24	30	26	24	24	22	22	30
TWK-43	39	23	28	27	23	25	27	24	20	25	26	38	27	25	22	28	19	33	24	26
TWK-44	22	21	27	25	24	20	25	19	21	27	31	24	21	36	28	23	20	22	20	35
TWK-45	21	21	25	21	24	22	24	19	17	23	22	23	22	27	22	23	22	22	23	29
TWK-46	25	22	32	29	26	26	27	23	21	23	24	25	22	27	23	23	26	19	24	27
TWK-47	19	27	18	20	24	22	19	18	16	26	21	19	22	20	20	26	16	20	28	22
TWK-48	25	24	31	26	29	24	26	22	21	28	24	25	24	32	24	29	20	26	25	31
ORK1/KCNKO	22	22	28	25	22	21	20	22	25	25	24	24	20	23	19	22	25	21	20	29
dTask-6	26	24	56	22	28	23	28	22	21	26	28	25	27	29	24	25	27	21	27	25
dTask-7	23	25	56	22	28	25	31	21	23	30	26	25	24	28	23	28	27	23	24	23
Sandman	22	21	26	25	25	24	24	23	21	24	24	23	23	24	23	26	23	26	23	29
CG10864	22	23	24	21	26	23	26	21	20	22	23	21	20	24	23	26	20	23	22	27
CG34396	28	22	30	27	31	29	29	25	22	29	28	28	26	28	26	28	22	24	27	29
CG43155	33	23	31	26	32	27	32	24	22	34	25	33	25	27	26	34	24	26	26	31
Galene	22	26	32	25	27	29	28	27	23	29	24	23	25	26	25	29	26	27	26	30

	TWK-20	TWK-21	TWK-22	TWK-23	TWK-24	TWK-25	TWK-26	TWK-28	TWK-29	TWK-30	TWK-31	TWK-32	TWK-33	TWK-34	TWK-35	TWK-36	TWK-37
TWK-21	25																
TWK-22	26	72															
TWK-23	27	26	25														
TWK-24	24	27	27	27													
TWK-25	24	25	24	20	21												
TWK-26	24	27	28	23	23	34											
TWK-28	29	25	25	27	21	24	28										
TWK-29	19	27	26	26	22	20	24	19									
TWK-30	24	24	25	28	21	22	21	31	23								
TWK-31	22	30	30	28	31	27	28	29	24	26							
TWK-32	21	24	24	29	23	23	21	28	21	29	25						
TWK-33	22	27	26	23	19	55	33	26	23	22	26	23					
TWK-34	22	27	26	25	20	53	32	25	23	20	27	24	79				
TWK-35	29	35	34	29	27	19	26	29	22	27	34	26	21	22			
TWK-36	25	27	28	25	24	49	33	25	25	21	28	22	58	53	22		
TWK-37	22	28	27	24	19	21	24	22	20	21	26	22	22	23	25	21	
TWK-39	26	29	26	25	21	22	29	26	27	24	24	24	24	26	28	23	24
TWK-40	29	24	23	31	22	26	28	40	24	32	33	28	25	26	27	27	23
TWK-42	26	24	26	24	28	19	25	26	21	24	33	23	20	22	31	22	23
TWK-43	24	25	24	33	20	23	22	28	27	24	24	21	25	25	25	23	24
TWK-44	23	29	31	25	30	23	27	25	22	22	35	23	26	23	33	28	25
TWK-45	22	27	27	23	22	31	72	23	23	19	27	21	30	31	24	32	24
TWK-46	26	26	24	27	24	22	25	24	19	22	26	21	20	22	26	26	23
TWK-47	20	19	22	21	17	20	20	26	20	20	23	17	20	19	21	23	19
TWK-48	25	32	30	25	27	27	32	28	26	26	29	22	29	29	31	30	25
ORK1/KCNKO	22	24	21	24	20	22	22	23	18	19	23	21	20	22	22	25	20
dTask-6	48	25	22	29	27	23	26	28	23	24	28	22	22	23	27	26	27
dTask-7	44	25	23	28	25	21	26	27	22	24	25	21	22	22	27	24	23
Sandman	26	27	24	23	22	25	28	25	26	23	26	19	23	24	27	24	27
CG10864	24	25	22	22	22	20	21	28	23	23	27	21	19	22	27	22	23
CG34396	28	30	28	28	24	28	30	30	27	25	32	28	29	27	35	26	28
CG43155	28	28	28	30	30	24	29	34	24	25	31	27	24	23	29	27	27
Galene	29	26	26	27	23	25	30	31	24	24	31	23	25	26	27	26	29

	TWK-39	TWK-40	TWK-42	TWK-43	TWK-44	TWK-45	TWK-46	TWK-47	TWK-48	ORK1/KCNKO	dTask-6	dTask-7	Sandman	CG10864	CG34396	CG43155
TWK-40	26															
TWK-42	24	27														
TWK-43	23	24	21													
TWK-44	25	25	35	19												
TWK-45	26	25	23	22	28											
TWK-46	24	25	25	24	26	22										
TWK-47	20	23	19	21	18	17	18									
TWK-48	38	28	28	25	28	27	24	22								
ORK1/KCNKO	22	24	22	23	24	20	28	17	20							
dTask-6	32	29	28	24	27	25	31	18	33	25						
dTask-7	27	29	26	23	27	24	29	20	28	25	64					
Sandman	37	26	22	26	25	27	25	17	37	21	28	25				
CG10864	30	23	23	23	24	21	24	20	34	19	22	25	37			
CG34396	30	30	29	26	30	31	26	21	33	26	32	27	33	27		
CG43155	30	33	27	30	27	31	25	22	31	24	30	30	29	27	35	
Galene	33	30	27	24	26	27	26	21	39	24	33	31	38	35	31	34

TWIK1, NP_002236 ; TWIK2, NP_004814 ; TRAAK, NP_201567 ; TREK1, NP_001017425 ; TREK2, NP_612190 ; TASK1, NP_002237 ; TASK3, NP_001269463 ; TASK5, NP_071753 ; TASK2, NP_003731 ; TALK1, NP_001128578 ; TALK2, AAH25726 ; TRESK, NP_862823 ; THIK1, NP_071337 ; THIK2, NP_071338 ; KCNK7, NP_203133 ; EGL-23, NP_001255776 ; UNC-58, NP_741880 ; SUP-9, NP_494333 ; TWK-1, NP_492054 ; TWK-2, NP_494786 ; TWK-3, NP_495727 ; TWK-4, NP_001343566 ; TWK-5, NP_001021895 ; TWK-6, NP_497973 ; TWK-7, NP_498903 ; TWK-8, NP_001023596 ; TWK-9, NP_501724 ; TWK-10, NP_001300134 ; TWK-11, NP_001343632 ; TWK-12, NP_505731 ; TWK-13, NP_506091 ; TWK-14, NP_001256414 ; TWK-16, NP_508526 ; TWK-17, NP_001024466 ; TWK-18, NP_509516 ; TWK-20, NP_510284 ; TWK-21, NP_510654 ; TWK-22, NP_510655 ; TWK-23, NP_001257229 ; TWK-24, AAC32865 ; TWK-25, NP_502170 ; TWK-26, NP_508522 ; TWK-28, NP_508732 ; TWK-29, NP_001021467 ; TWK-30, NP_492381 ; TWK-31, NP_001022883 ; TWK-32, NP_506416 ; TWK-33, NP_001309463 ; TWK-34, NP_506906 ; TWK-35, NP_508031 ; TWK-36, NP_507485 ; TWK-37, NP_491810 ; TWK-39, NP_001076639 ; TWK-40, NP_001255207 ; TWK-42, NP_507483 ; TWK-43, NP_872137 ; TWK-44, NP_509942 ; TWK-45, NP_001122742 ; TWK-46, NP_741678 ; TWK-47, NP_001309491 ; TWK-48, NP_001022681 ; ORK1/KCNK0, NP_511112 ; dTask-6, NP_001262539 ; dTask-7, NP_649891 ; sandman, NP_610349 ; CG10864, NP_650726 ; CG34396, NP_001097392 ; CG43155, NP_572720

Supplementary Figure 2

TM1 helix

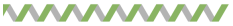

```
TWIK1         19 ------SAWCFGFLVLGYLLYLVFGAVVFSSVELPYEDLLRQEL------R--------------------K--------------LKRRFL--------
TWIK2          5 --------ALLAGALAAYAAYLVLGALLVARLEGPHEARLRAEL------E--------------------T--------------LRAQLL--------
TRAAK          5 ---------TLLALLALVLLYLVSGALVFRALEQPHEQQAQREL------G--------------------E--------------VREKFL--------
TREK1         60 --------KTVSTIFLVVVLYLIIGATVFKALEQPHEISQRTTI------V--------------------I--------------QKQTFI--------
TREK2         75 --------KTVVAIFVVVVVYLVTGGLVFRALEQPFESSQKNTI------A--------------------L--------------EKAEFL--------
TASK1          4 ------QNVRTLALIVCTFTYLLVGAAVFDALESEPELIERQ------RLE--------------------LRQQE-------LR-ARYNLS--------
TASK3          8 ----------TLSLIVCTFTYLLVGAAVFDALESDHEMREEE------KLK--------------------AEEIR-------IK-GKYNIS--------
TASK5          6 --------VRAAGLVLCTLCYLLVGAAVFDALESEAESGRQR------LLV--------------------QKRGA-------LR-RKFGFS--------
TASK2          4 ---------RGPLLTSAIIFYLAIGAAIFEVLEEPHWKEAKKNY------Y--------------------T--------------QKLHLL--------
TALK1          9 -----CWGGRVLPLLLAYVCYLLLGATIFQLLERQAEAQSRDQF------Q--------------------L--------------EKLRFL--------
TALK2         19 -------VPGTVLLLLAYLAYLALGTGVFWTLEGRAAQDSSRSF------Q--------------------R--------------DKWELL--------
TRESK         20 -------LFPGLCFLCFLVTYALVGAVVFSAIEDGQVLVAADDGEFEK-----------------------------------FLEELCRILNCSE----
THIK1         19 ---------RFLLLAALIVLYLLGGAAVFSALELAHERQAKQRW------E--------------------ERLAN--------------FSRGH-----
THIK2         38 ---------RFVLLAALIGLYLVAGATVFSALESPGEAEARARW------G--------------------ATLRN--------------FSAAH-----
KCNK7          6 ------PWSRYGLLVVAHLLALGLGAVVFQALEGPPACRLQAEL------R--------------------A--------------ELAAFQ-------A
EGL-23        19 ---KATPLFVHFLMIVSVGAYAIFGALVMRSLESRTVTSIEKKTDVHRRHVNLTNFQHPPTP----ITLEQRHRRRRRHNETALEDHLSEKLSREKRAAA
UNC-58       139 ----IKILTPHVILVSVLIGYLCLGAWILMLLETRTELLARSKKL--VRLT--------------------NLMSN-------FTAESWKMLNNAQHGVS
SUP-9          6 --------IRTLSLIVCTLTYLLVGAAVFDALETENEILQRK------LVQ--------------------RVREK-------LK-TKYNMS--------
TWK-2         32 -----KLALPHIVLVVCVCIYATIGAWIFYTLESPNEDRLKETGR--KTIA--------------------EMRSN-------LIYKINN----------
TWK-3         36 -----GPLALHTGLVLSCVTYALGGAYLFLSIEHPEELKRREKA----------------------IREFQDLKQQ-------FMGNITS----------
TWK-4        130 ------LALFHLVLIFATVAYIIAGAYLFTKIEHQAELDRYQSYH--------------------------TIYRN-------FINNLYQSSNRS-----
TWK-6         35 ------NVVVCLSAAITLLVFNLIGAGIFYLAETQNSSESLNENS--EVSK--------------------CLHNL-------P----------------
TWK-7        159 ----AKLVLPHVALVLLTCTYTVIGALIFYSVEQPHEQMMKEQQL--KLIY--------------------TRQNE-------FVDDLIRLAAGNE----
TWK-8         86 -------HIKYLFPLIFIMFYMLIGAIIFYLLESGTAEDAANEED--YKYK--------------------RERRL-------LLLRMEELVQ-------
TWK-9         14 ----ASTLFVHVALIAGVAVYTVFGALSMQWLESPDRVRALLKRE----LKPVESLPPPPSI----SGLPDRITRVYLGEELAILDP-------------
TWK-10        36 -------GGLHVGLILLCILYVHFGALFFMYSESPEEKYFLENLL--KLTFVSDSHEKSQRILRRVEKRYDILRET-------FLENVNRVKLEDF----
TWK-11        77 ------------KLLIIIGLYSFIGAHIFMYLEVPTDLEAREDGF--HQRK--------------------IAREV-------MVLNLRAIFY-------
TWK-12        12 ----NLIRLRSYYKFLLLIAYTAFGAWLFRTYELQADIKRRSVFG--NTTN--------------------LVRRQ-------LAERWIEMHK-------
TWK-13        50 --RWFRLALPHFGLVLLSIGYTLIGALCFHHYEKPYEQQLRNETS--RRIG--------------------ELKNR-------VMDQLWRMSNNG-----
TWK-14        49 --------ARFVLICIILIVYLAFGAILFHWLEWENEVDERIAI------D--------------------NRMAD--------------YQKVY---CK
TWK-16        22 ------PFTLHCSLLMLVLLYSFLGGFIFDRIETNAHAEMKRNERINRTAC---------------------------------VSQILHSIHRWS----
TWK-17       136 ---LLKILLPHVGLNVLLLSYIAMGATVFIWLEADHELEGRKAKV--KHVF--------------------DIYSQ-------IMNETIALTNNQSDQAT
TWK-18        20 ---------KGLLPLIILVAYTLLGAWIFWMIEGENEREMLIEQQ--KERD--------------------ELIRR-------TVYKINQLQI-------
TWK-20        11 ----------ALLLILSTFTYLLFGAMVFDKLESEKDTWVRD------EIE--------------------RITDR-------LK-HKYNFS--------
TWK-21        33 -----LLGIRYIMLILIILGYACLGGYMFQALEYDQQQLELEAEK--RVRL--------------------SESSL-------LAVNLLEH---LKQMNC
TWK-22       126 -----LLGIQYMLLALMILGYACLGGYIFLTLEYDQQQLDLEVEK--QVRL--------------------SESTL-------LAENLLKY---LKQWNC
TWK-23         9 ------IALGHLALYCFVVCYVFAGAWVFHQLEGENETELHDKQR--EYAM--------------------NLKKD-------VIAKLATTENVAE----
TWK-24       117 ----------HIAWLVVLFSFSLFGGVIFSAIEGGYETTQLIKKF--EHEK--------------------DVYER-------RKIYQEQLFQRLREIEH
TWK-25       136 ------IGFRHVCMLLLVLLYTLLGAALFFSIESRHEHETMHFHK--RKLD--------------------RIIYE-------IAQTLELEVLDPMKLTN
TWK-26        82 ------FGIRHVTLIALLVGYVFLGGFMFEKLESPRELEDLKETI--VLMQ--------------------GIIDE-------ETTDIINVTLSTNGTDR
TWK-28        57 -----KRILPHVGLVILLFLYLIAGAFLFRYLEAPKELETRNHEL--TTIL--------------------GLRDE-------FQDHIWNITQDSDNR--
TWK-29        48 ------AIAVNGFIIVFLIIYTTIGGFIFLNFEFEYQQYMKQNATLEKRLC---------------------------------IESLLNRDNRLR----
TWK-30        15 -------LSLHVLLIGSVVLYIILGAIVFQMLEGEHLDALKKDHM--AKIE--------------------QNAKD-------YVDKLWSVAKRDR----
TWK-31       142 --------LDRISASVLLVLYSFLGAWVFYLFEHDYEREVKLKER--IDLR--------------------MLRND-------TFQRISSMVF-------
TWK-32        32 ------LALPHITLLVVSILYAVFGGWMLTLIKYSQQTT---HQA--IMFD--------------------QTRQN-------FAKRLALIGNKEE----
TWK-33       211 -----QIGFRHFSVVILVLLYTLLGAVMFWTVESRHEKAKTLDHV--NNLE--------------------HLLDR-------LAENITESVNNINTTTT
TWK-34        90 ------IGLRHIMVALMVLSYTIFGAFMFWTVESRNERAVTLERV--TNLE--------------------NLLNI-------LATNITEIVNNPNTTTT
TWK-35       144 -----------AVLIIVFLIYCISGGLVFWLIEEPYQSELRDAWQ--HKIE--------------------NNRT-----------------ARVDAMMK
TWK-36       166 ------FGLRYIALLVLALIYTLLGATVFYLIEGSNEKSRLHVRE--QNLD--------------------KLLDE-------LATVLSEAVNDPEQSSE
TWK-37        60 -------KFNTIIAFVVLVAYIIGGALLFWQIESRSDMNINEFKR--NIRQ--------------------KSI---------------HIYNSMKGSKC
TWK-39        19 ---ATIFTITHVGLCFLVALYAVAGAFMFQAVEYPYELGLQG------KVK--------------------NASLK-------VVDDIYRFINRKNV-IE
TWK-40        10 ------IILAHVSLIVLSVVYVGFGAFLFYQLEQPNEVEVRARNI--ERFN--------------------IHKRQ-------MIEHLWEMRESG-----
TWK-42         9 --NYERYHLHHLVKIGVLIVYSFLGAGLFVLCEAENEKSLKHEDN--MRVL----------------------RTS-------IA----AKQVFVQRLQN
TWK-43        37 ------PYLLNAGIVLFVGIYVYAGALLIQYIERIPVKVEESF----------------------------RLRRNS-QD---------------DMPII
TWK-44       212 -------RVHYLVPIILLIAYSVLGGLIFWSIERPNEEIMLADKR--NYIA--------------------ALTDD-------LVEVLMQIHNRLIDFNR
TWK-45        47 ------FGIRHITLISILAAYICLGGFLFQKLESPREIEELQETL--KSMN--------------------EIIKN-------ETMDIIKITLTTNGEDR
TWK-46        27 ----------ILVGLGVAVVYLFVGAIVFVRIEYPLEKIEREAY------L--------------------D--------------YQNQWR-------D
TWK-48        20 ---IIAFFFSHIGLCALVVGYALLGAVIFKAVEGPHEAEIQE------LVK--------------------SAREK-------AVDVVWNATFRVNR-LD
ORK1/KCNK0     1 -----MSPNRWILLLIFYISYLMFGAAIYYHIEHGEEKISRAEQ------R--------------------KAQI---------A-INEYLL--------
dTask-6        1 ---MKKQNVRTISLIVCTFTYLLVGAAVFDALESETEKRRWE------ALQ--------------------DAEDM-------II-RKYNIS--------
dTask-7        1 --MMKRQNVRTLSLVVCTFTYLLIGAAVFDSLESPTEAKRWE------FLQ--------------------TVKNN-------FV-RKYNVT--------
sandman       26 ---FTAFMFSNVGIILLVTFYIIGGAFIFQSIEIFEYERLKSEKPHRFIAR--------------------NFSGE-------CLSRIWELTAENISFFD
CG10864       42 --NFVTFMCTQVGVGALIVIYAICGAFAFMHIERQFVDETAG------HVM--------------------ELRQN-------CSQQLWSITEQHNI-ID
CG34396      111 -------WFTHIFLILILALYNIGGAVVFRTIEVRHANQE--------KLV-------------------------------------------VQDQQK
CG43155       61 ------SALNHIGLLVSLSIYCGVGGLIFRHLERPAEVERLSHLK--DIVK--------------------THRER-------FLHTILNNTEVH-----
Galene        31 SWKVLTCIVSHVLLVLLVVSYCVGGAYLFQHLERPHELEVKR------DIQ--------------------NLRVN-------LTENIWLLSDDAVV-LR
```

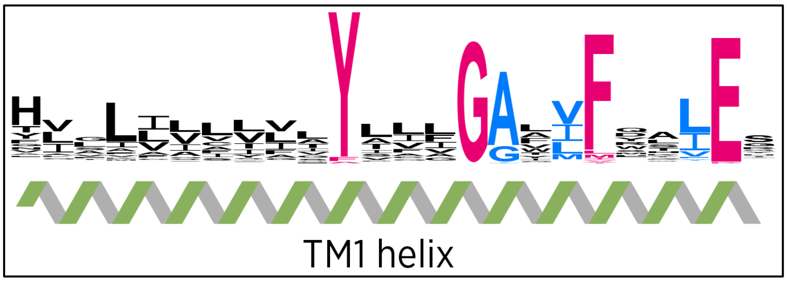

Supplementary Figure 2 - Clustal Omega alignment of human, *C. elegans*, and *D. melanogaster* two-pore domain potassium channel sequences.

Genbank identifiers for aligned sequences are listed below. N- and C-terminal cytoplasmic portions were truncated before alignment. Cartoon representations on top mark the four transmembrane helix regions (TM1, TM2, TM3, and TM4), two pore helix domains (Ph1 and Ph2), and two selectivity filters (SF1 and SF2). Sequence logos were generated using WebLogo 3 (Crooks et al., 2004). Highly conserved residues are indicated in *hotpink* and well conserved residues in *marine*, as in Figure 1.

TWIK1, NP_002236 ; TWIK2, NP_004814 ; TRAAK, NP_201567 ; TREK1, NP_001017425 ; TREK2, NP_612190 ; TASK1, NP_002237 ; TASK3, NP_001269463 ; TASK5, NP_071753 ; TASK2, NP_003731 ; TALK1, NP_001128578 ; TALK2, AAH25726 ; TRESK, NP_862823 ; THIK1, NP_071337 ; THIK2, NP_071338 ; KCNK7, NP_203133 ; EGL-23, NP_001255776 ; UNC-58, NP_741880 ; SUP-9, NP_494333 ; TWK-2, NP_494786 ; TWK-3, NP_495727 ; TWK-4, NP_001343566 ; TWK-6, NP_497973 ; TWK-7, NP_498903 ; TWK-8, NP_001023596 ; TWK-9, NP_501724 ; TWK-10, NP_001300134 ; TWK-11, NP_001343632 ; TWK-12, NP_505731 ; TWK-13, NP_506091 ; TWK-14, NP_001256414 ; TWK-16, NP_508526 ; TWK-17, NP_001024466 ; TWK-18, NP_509516 ; TWK-20, NP_510284 ; TWK-21, NP_510654 ; TWK-22, NP_510655 ; TWK-23, NP_001257229 ; TWK-24, AAC32865 ; TWK-25, NP_502170 ; TWK-26, NP_508522 ; TWK-28, NP_508732 ; TWK-29, NP_001021467 ; TWK-30, NP_492381 ; TWK-31, NP_001022883 ; TWK-32, NP_506416 ; TWK-33, NP_001309463 ; TWK-34, NP_506906 ; TWK-35, NP_508031 ; TWK-36, NP_507485 ; TWK-37, NP_491810 ; TWK-39, NP_001076639 ; TWK-40, NP_001255207 ; TWK-42, NP_507483 ; TWK-43, NP_872137 ; TWK-44, NP_509942 ; TWK-45, NP_001122742 ; TWK-46, NP_741678 ; TWK-48, NP_001022681 ; ORK1/KCNK0, NP_511112 ; dTask-6, NP_001262539 ; dTask-7, NP_649891 ; sandman, NP_610349 ; CG10864, NP_650726 ; CG34396, NP_001097392 ; CG43155, NP_572720 ; Galene, NP_612084.

Supplementary Figure 2

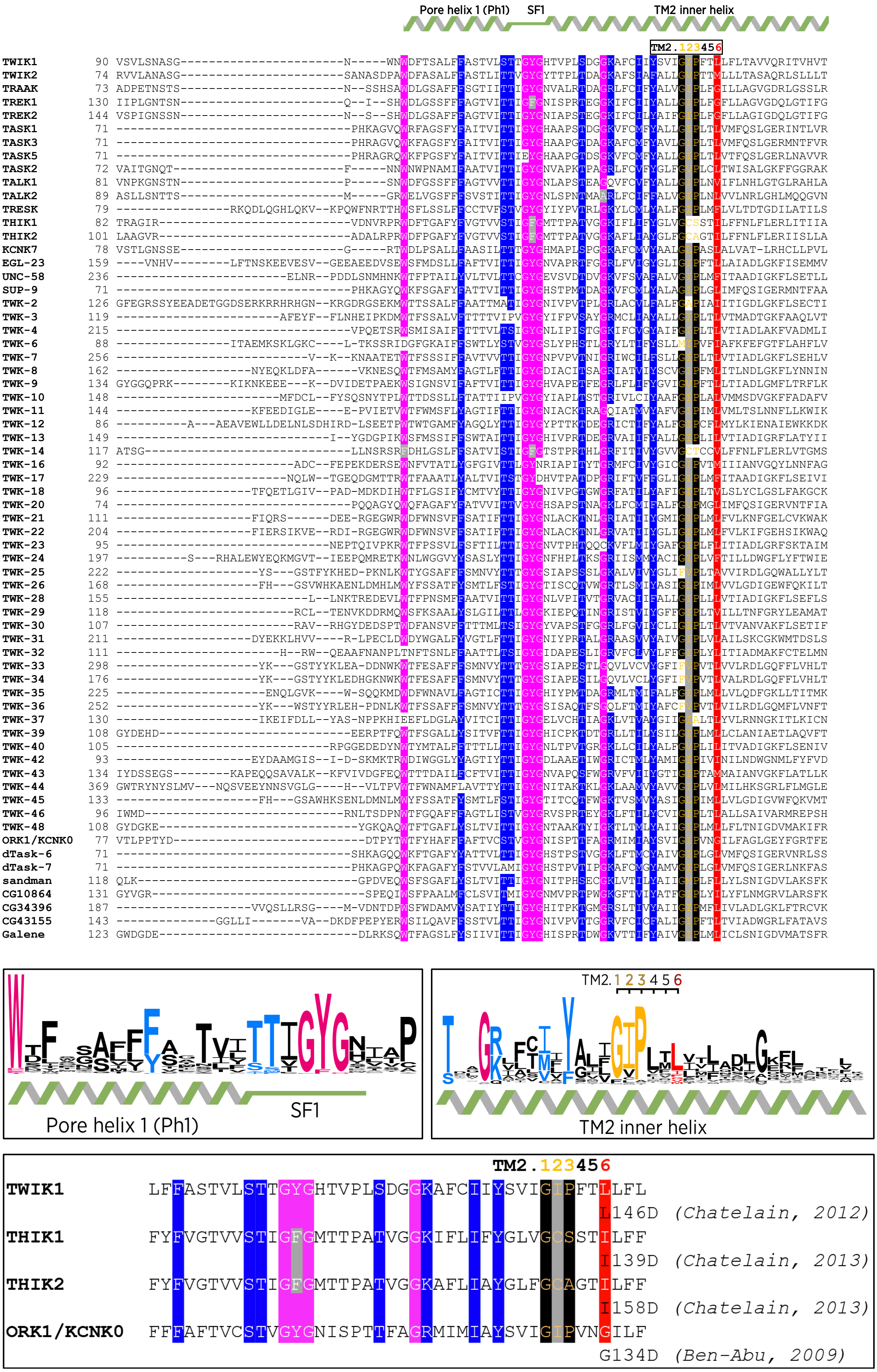

Previously published gain-of-function mutants of TM2.6 (see manuscript for references).

Supplementary Figure 2

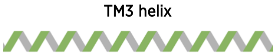


```
TWIK1       179 --------------------------------------------------------------------VAIVHAVLLGFVTVSCFFFIPAAVFSVLED--
TWIK2       168 --------------------------------------------------------------------AACWHLVALLGVVVTVCFLVPAVIFAHLEE--
TRAAK       167 --------------------------------------------------------------------VRVLSAMLFLLIGCLLFVLTPTFVFCYME---
TREK1       221 --------------------------------------------------------------------IRIISTIIFILFGCVLFVALPAIIFKHIE---
TREK2       236 --------------------------------------------------------------------IRVISTILFILAGCIVFVTIPAVIFKYIE---
TASK1       155 ---------------------------------------------------------------------SMANMVLIGFFSCISTLCIGAAAFSHYE---
TASK3       155 ---------------------------------------------------------------------SMENMVTVGFFSCMGTLCIGAAAFSQCE---
TASK5       155 ---------------------------------------------------------------------STENLVVAGLLACAATLALGAVAFSHFE---
TASK2       158 --------------------------------------------------------------------AQITCTVIFIVWGVLVHLVIPPFVFMVTE---
TALK1       167 --------------------------------------------------------------------LQVLGLALFLTLGTLVILIFPPMVFSHVE---
TALK2       179 --------------------------------------------------------------------LA---GSGALLSGLLLFLLLPPLLFSHME---
TRESK       268 ----------------------------------------------------------N-LDEVGQQVERLDIPLPIIALIVFAYISCAAAILPFWET--
THIK1       182 -------------------------------------------------CEVDSLA--------GWKPSVYYVMLILCTA-SILISCCASAMYTPIE---
THIK2       201 -------------------------------------------------SEADSLA--------GWKPSVYHVLLILGLF-AVLLSCCASAMYTSVE---
KCNK7       166 --------------------------------------------------------------------AALLQAVALGLLVASSFVLLPALVLWGLQG--
EGL-23      294 ------------------------------------------------------------E-VSEEEDDLTETEATSLFILFLVYIAFGGFMLAAYEP--
UNC-58      348 ----------------------------------------------------------D-ILGVDGTEEKLWFPIGAYVSCICIYCSIGSAMFITWER--
SUP-9       155 ---------------------------------------------------------------------SSDLIIFCTGWG-GLLIFGGAFMFSSYE---
TWK-2       265 ----------------------------------------------------------I-LDMDMDEIDKSEVPVLMVFTIILLYI-AFGGILFSILE--
TWK-3       205 --------------------------------------------------------------------------AAIFVCLLFAYPLVVGFILCST-S--
TWK-4       285 ------------------------------------------------------------------TEDPKTGRQLLVLVFLLGYMTISACVYTILEP--
TWK-6       191 ----------------------------------------------------------E-TPSNSL--QHDYLIFLSSLLLCSISLLSSSALFSSIE---
TWK-7       356 ----------------------------------------------------------H-GMGHDMNIEEKRIPAFLVLAILIVYT-AFGGVLMSKLE--
TWK-8       302 ----------------------------------------------------------E-DIEDEEERSMPRMSVKVALGITVGWIFFCSALFKLWE---
TWK-9       302 ----------------------------------------------------------E-E-EPENNEPRKTEESIALGITFTCYLVAGAKILSVYEP--
TWK-10      240 ------------------------------------------------------------------KIQNITAFMVVLLILLVAYSVIGGIAYSKI-V--
TWK-11      288 ----------------------------------------------------------E-DDE-EEEEIHQDPPVLSTLIATVAWIILSAAVFCLFE---
TWK-12      228 ----------------------------------------------------------E-DPEAAEERKKKPFPIPIAIIMLIIWICFSASMFCIWED--
TWK-13      325 ----------------------------------------------------------E-QIQFDPSNHEKRVSVLFILLIMLGYV-AGGAYIVRWWE--
TWK-14      225 -------------------------------------------------SCGGHMD--------NWRPSVYKVFFILFSM-CLVLITASAGIYSVVE---
TWK-16      188 ----------------------------------------------------------------IYKESSIQVTSLALLCVFLIYVAVGALLLPLLNG--
TWK-17      351 ----------------------------------------------------------D-DEECEDEEDRLQLPIASYFTLIIGYCCVGSLLFNTFEK--
TWK-18      213 ----------------------------------------------------------E-KTE-NNDDDLLSFPISGLLLITVIWVIFCAVLFTFLE---
TWK-20      162 ---------------------------------------------------------------------TPTHLLMVSLTIGFMVIVSGTYMFHTIE---
TWK-21      220 ----------------------------------------------------------F-FEVPEDDKEDTTFQLRWGLLVIVLFVVLCSFVVSFWE---
TWK-22      300 ----------------------------------------------------------E-SGISEDEEQITTFPVKWALFIVFFFMVVCSLIVSFWE---
TWK-23      176 -------------------------------------------------------------KQSDEHLLREIAEVLLVAGLFVVFIAIGSAVIPLWEN--
TWK-24      348 ----------------------------------------------------------L-DVDVPMGEQVQTVPIKSAAIFFFLWIMISAFIVRLWEY--
TWK-25      312 ----------------------------------------------------------G-FVDKLDEDEIITLPIKFSVSVMILYLLSATMFIFEYDELS
TWK-26      259 ----------------------------------------------------------K-QPVNRKKN-EILLPMWLALFLVLAYILICTLTIKMFDHNE
TWK-28      275 ----------------------------------------------------------D-SDSEDSAGDELRIPVFMVLLVLLAYT-AIGGFLFQSWE--
TWK-29      205 --------------------------------------------------------------------EDENVSGSTLFFIVLVYLILGATMIPLMSG--
TWK-30      215 ------------------------------------------------------------EEEILDRVRLVRFPPLTVFFFVFVYG-CIAAWVVRYWE--
TWK-31      327 ----------------------------------------------------------A-DPEKKPEVESRTIPIWLALLICVVYVAGCSSLFLLWET--
TWK-32      230 ----------------------------------------------------------F-LWTHLEHAQFVEVPFLLVIGILLLYI-GLSSWIISWVE--
TWK-33      388 ----------------------------------------------------------G-N-KHVDEDEIISLPIKACLLLLASYLGACTIFIYFYDELS
TWK-34      267 ----------------------------------------------------------G-D-TSIDVNEIIKIPIKACLLLLALYLAFCTIFIHVFDELS
TWK-35      327 ------------------------------------------------------------ERQERHDLDIFDLPLPVGIALIVTWIFICSFVLSVWDH--
TWK-36      342 ----------------------------------------------------------G-K-REVDEDEIIQLPIKFCMTILIAYLLLCTTFVYLYDAVM
TWK-37      226 ----------------------------------------------------------------------YKMTVLKAFILLVTFWGFGALAIAVYE---
TWK-39      427 SA-RELR----EKEMMMMNSLNHKQPSMDSSTSRRLRDIDGRSYRSDRSERSDEMSLHSLRRNGHRNQEKMPV--SVGICIVFAFISGGAWLFSWWE---
TWK-40      207 -------------------------------------------------------------LNLDHLENYISIPIFLIVAILLSYI-TFGAVVLSMWE--
TWK-42      213 ----------------------------------------------------------P-SP--QNPNGTRPIPLLLVLIVLFFWMIQCVAYFAYFE---
TWK-43      277 ----------------------------------------------------------E-E-AEEEVEESDVTETIVLFVAFLVYIIAGSLLMSYYEE--
TWK-44      482 --------------------------------------------------------------RNTGEDRISEMPLILAIGVAFGWMFLCAAIFLRFEK--
TWK-45      241 ----------------------------------------------------------K-QSIEIKRK-ETLLPMWLAMLVVFTYIIICTLTILLFDDNE
TWK-46      186 --------------------------------------------------------------------IQLIHVGVVFASLLLFVFAIPAWVFSSIET--
TWK-48      247 M--------------EQL----------------------------------------EVRETAAAQLESMTVPISLVVFTMLGYLGVGTTIFKVWE---
ORK1/KCNK0  172 --------------------------------------------------------------------GLITTVVIALIPGIALFLLLPSWVFTYFE---
dTask-6     155 ---------------------------------------------------------------------SEVDLICVVTTLSSLTIAGGAAAFSKFE---
dTask-7     156 ---------------------------------------------------------------------TEMNLMLATGMLSSIIITTGAAVFSRYE---
sandman     264 ------------------------------------------QYTESDS---------DIEREIRGSTDEITVPVTVCVFVMVGYILWGALLFGRWE---
CG10864     221 ----------------------------------------------------------ALEGGVGMTRKKVIVPSTACLWVIFFYVLTGTVMFANWE---
CG34396     340 ----------------------------------------------------------P-YPETFEVDDEFNLPVSVASLLLITYILLGSFGFLMMEP--
CG43155     234 ------------------------------------------------------------------FIGKTWFYAILAVGFLGVYLAAGAGLLLLWED--
Galene      552 SRSREPKRNRRRERAERLPPSPRIMSPMGFPVQRQIRRRPSYDYDDDD-----SMYGDEYGDYGDLLPKDRPVPIWLCVFLVVSYILGGAVLFAYWE---
```

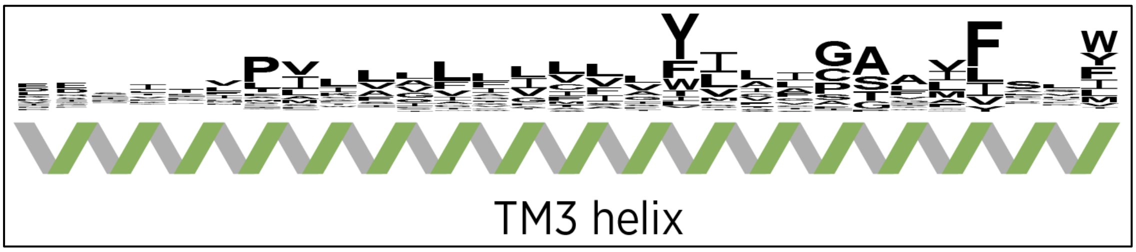

Supplementary Figure 2

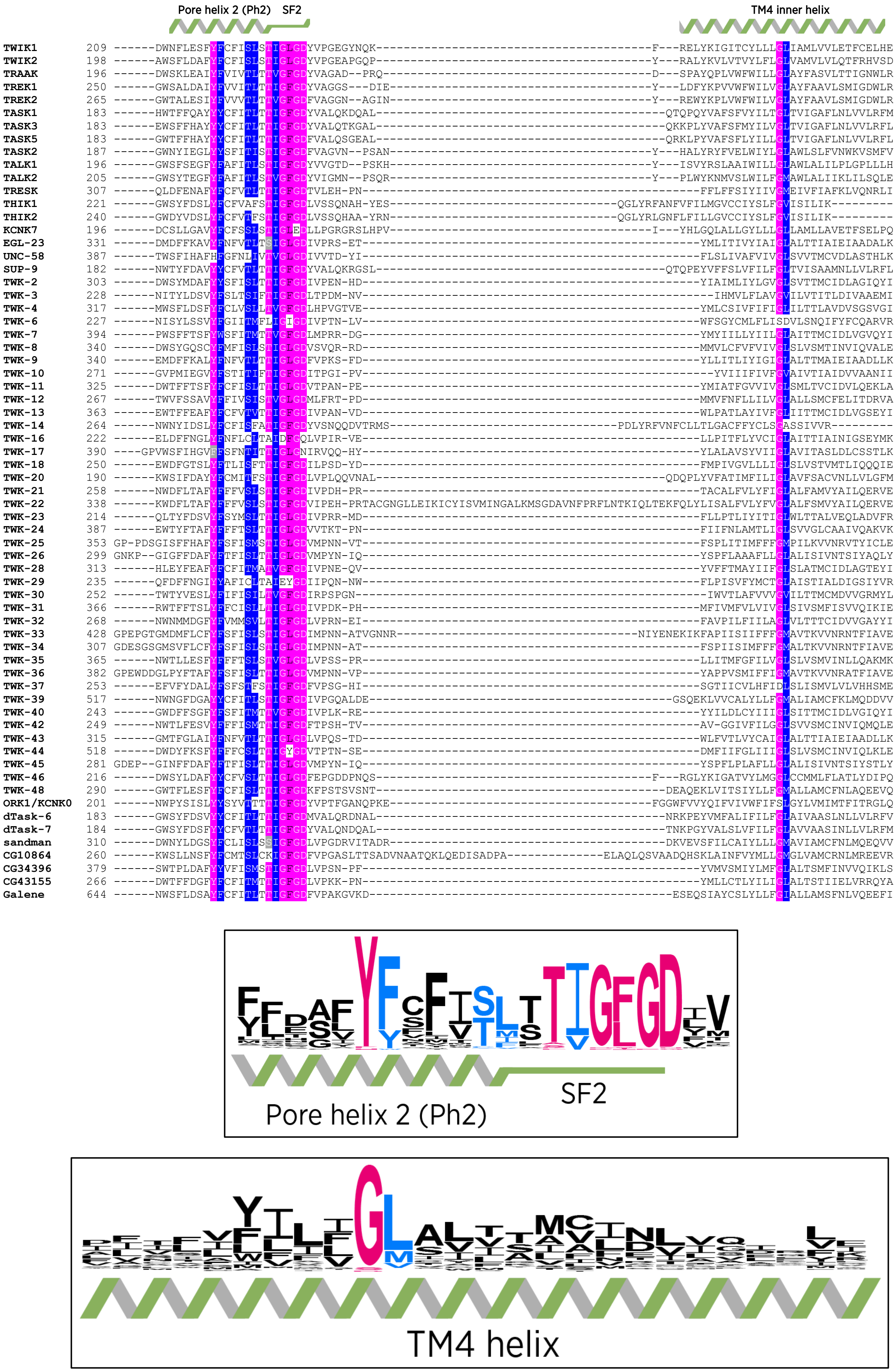

Supplementary Figure 3

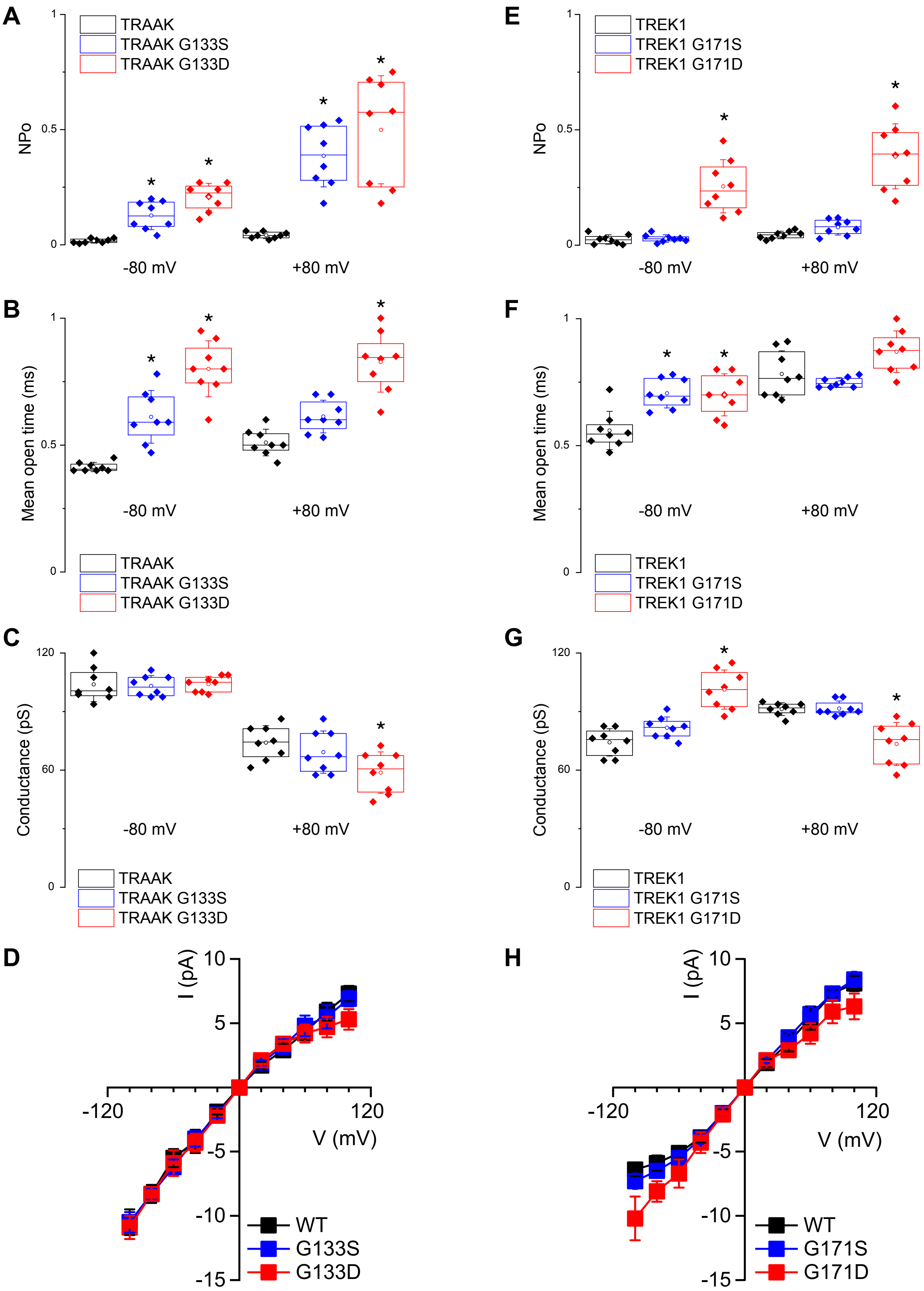

Single channel properties of TRAAK and TREK1 TM2.6 mutants

(A) TRAAK, TRAAK G133S, TRAAK G133D, (E) TREK1, TREK1 G171S, and TREK1 G171D channel activity determined by NPo analysis at -80 and +80 mV.

(B), (F) Mean open times were obtained from channel openings recorded at -80 mV and +80 mV.

(C), (G) Unitary conductances obtained from channel openings recorded at -80 mV and +80 mV.

(D), (H) Current-voltage relationships of single channels.

Center lines, medians; open circles, means; box limits, 25th and 75th percentiles; whiskers, standard deviation. Kruskal-Wallis, Dunn's multiple comparison test, * $p < 0.05$.

Supplementary Table 1: Two-electrode voltage-clamp experiments (Figure 1B)

Channel	TM2.6	cRNA	Day	E_{rev} (mV)	I (µA) at 0 mV
hTWIK1	WT	10 ng	1	-38.1 ± 2.4	0.2
	L146N			-88.4 ± 1	2.7 ± 0.2
rTWIK2	WT	10 ng	1	-33.1 ± 1.1	0.1
	M135D			-66.4 ± 2.2	0.4
hTASK1	WT	0.8 ng	3	-63.4 ± 2.5	0.7 ± 0.1
	L122N			-71.0 ± 1.1	13.4 ± 2
hTASK3	WT	50 pg	1	-73.4 ± 4.3	1.8 ± 0.3
	L122N			-77.6 ± 2.5	5 ± 0.8
mTREK1	WT	1 ng	1	-66.1 ± 1.5	0.7 ± 0.1
	G171D			-72.0 ± 0.6	8.8 ± 0.8
mTREK2	WT	1 ng	2	-52.5 ± 2	0.5 ± 0.1
	G196D			-72.1 ± 0.9	1.7 ± 0.2
mTRAAK	WT	1 ng	2	-55.6 ± 1.5	0.3
	G133D			-65.8 ± 0.8	32.9 ± 7.2
mTRESK	WT	1 ng	1	-70.8 ± 1.1	0.9 ± 0.1
	F156N			-68.9 ± 2	24.3 ± 3.6
mTASK2	WT	5 ng	1	-87.5 ± 2.8	3.8 ± 0.6
	L127N			-90.3 ± 6.6	14.2 ± 1.6
hTALK1	WT	20 ng	1	-80.5 ± 3.9	1 ± 0.1
	V137N			-93.6 ± 2.2	2.9 ± 0.2
hTALK2	WT	20 ng	1	-42.4 ± 2.1	0.3
	L145N			-51.4 ± 3.5	1.4 ± 0.1
hTHIK2	WT	50 ng	2	-31.1 ± 5.1	0.1
	I158D			-83.3 ± 0.9	1
hTASK2	WT	10 ng	1	-77.4 ± 2.7	0.4
	L127N			-93.4 ± 0.6	6.8 ± 0.2
rTWIK2 LY	WT	10 ng	1	-84.3 ± 0.8	2.2 ± 0.1
	M135D			-79.9 ± 0.8	8.5 ± 0.2
hTWIK1 AA	WT	10 ng	1	-71.7 ± 4	0.4
	L146N			-85.1 ± 0.9	9.3 ± 0.4
hTHIK2 5RA	WT	15 ng	1	-31.2 ± 1.4	0.2
	I158N			-76.5 ± 1	1.7 ± 0.2

Supplementary Table 2: Strain list

Strain	Genotype	Description
JIP1658	*egl-23(bln309) V*	EGL-23-wrmScarlet
JIP1506	*egl-23(bln309bln334) V*	EGL-23(L229N)-wrmScarlet
JIP1559	*egl-23(bln309bln359) V*	EGL-23(L229S)-wrmScarlet
JIP1620	*unc-58(bln448) X*	UNC-58(F294S)
JIP1621	*unc-58(bln449) X*	UNC-58(F294T)
JIP1622	*unc-58(bln450) X*	UNC-58(F294N)
JIP1623	*twk-18(bln451) X*	TWK-18(V158N)
JIP1636	*twk-18(bln458) X*	TWK-18(V158S)
JIP1706	*twk-18(bln491) X*	TWK-18(V158T)
JIP1677	*sup-9(bln476) II*	SUP-9(L122S)

Supplementary Table 3: List of single strand oligonucleotides (DNA and RNA) for CRISPR/Cas9 genome engineering

	**Sequence (5' to 3')**	**Description**
**crRNA *unc-58***	gcggcagugauagacauuag	Use to generate UNC-58(F294x)
oPT271	cggaaaggttttctcagtagcattcgcgcttgttggta taccactAatgtCTatCacTgcCgccgatattggtaaa tttttatctgaaacattactccagtttgtgagct	Repair template (Ultramer) for UNC-58(F294S)
oPT273	tcggaaaggttttctcagtagcattcgcgcttgttggt ataccactAatgACAatTacTgcCgccgatattggtaa atttttatctgaaacattactccagtttgtgagctt	Repair template (Ultramer) for UNC-58(F294T)
oPT275	tcggaaaggttttctcagtagcattcgcgcttgttggt ataccactAatgAATatTacGgcCgccgatattggtaa atttttatctgaaacattactccagtttgtgagctt	Repair template (Ultramer) for UNC-58(F294N)
**crRNA *egl-23***	ugguauuccguuuacacugc	Use to generate EGL-23(L229x)
oSEM402	ttcgcctccaccatcatttctgatatgaattttccgag atctgcaattgcTagACTtgtaaacggaataccaatta gaccataaccaatgacaaatagacggccac	Repair template (Ultramer) for EGL-23(L229S)
oSEM356	ttcgcctccaccatcatttctgatatgaattttccgag atctgcaattgcTagGTTtgtaaacggaataccaatta gaccataaccaatgacaaatagacggccac	Repair template (Ultramer) for EGL-23(L229N)
**crRNA *twk-18***	uaaagacugagcacugugag	Use to generate TWK-18(V158x)
oSEM435	ctggatggggtcgttttgctactatcctttacgcattc attggaattccaTtGacaAACTtAagtTtGtactgtct tggtagtctttttgccaagggatg	Repair template (Ultramer) for TWK-18(V158N)
oSEM436	ctggatggggtcgttttgctactatcctttacgcattc attggaattccaTtGacaAGTTtAagtTtGtactgtct tggtagtctttttgccaagggatg	Repair template (Ultramer) for TWK-18(V158S)
oSEM437	ctggatggggtcgttttgctactatcctttacgcattc attggaattccaTtGacaACTTtAagtTtGtactgtct tggtagtctttttgccaagggatg	Repair template (Ultramer) for TWK-18(V158T)
**crRNA *sup-9***	cgcacuggcgggaauuccau	Use to generate SUP-9(L122x)
oNZ161	tggaactcctggaaaattttcagcaactcaccaatact ctgaaacatgataTTtccTaGtggaattcccgccagtg cgtagagcatgcagaaaacttttccgg	Repair template (Ultramer) for SUP-9(L122N)
oMG798	tggaactcctggaaaattttcagcaactcaccaatact ctgaaacatgatGCTGccTaGtggaattcccgccagtg cgtagagcatgcagaaaacttttccgg	Repair template (Ultramer) for SUP-9(L122S)

Supplementary Table 4: Genotyping primer combinations for *C. elegans* mutants

Allele	Primer	Sequence (5' to 3')	Description
unc-58 F294X	oTB522	cggggttagtaatatggatgaagg	338bp PCR product; verify by sequencing.
	oTB508	ggaccacccgttgattgtaa	
egl-23 L229X	oSEM357	cccaccattaaccgagtttg	487bp PCR product digested by FspBI; WT: 134/353bp Mutant: 104/134/249bp
	oSEM358	tcccagattataagcaggcaat	
twk-18 V158X	oSEM430	tgcggcgcttaagtatttg	673bp PCR product digested by RsaI; WT: 673bp Mutant: 384/289bp
	oSEM431	ggagatggggaaagacaaca	
sup-9 L122X	oMG850	caactgtgataacgacgatcgg	PCR oMG850-852-856: WT:986/324bp L122N&L122S:986bp PCR oMG850-852-855 WT:986bp L122N:986/324bp PCR oM850-852-853 WT:986bp L122S:986/324bp
	oMG852	caccgcgtcgaaatatgtcc	
	oMG853	cgggaattccaCtAggCAGC	
	oMG855	gcgggaattccaCtAggaAA	
	oMG856	gcgggaattccaTtGggACT	

Supplementary Table 5: List of plasmids

Plasmid/ gene	Primer	Sequence (5' to 3')	Isothermal Ligation with EcoRV digested pTB207 and	
pTB301/ *TWK-18 WT*	oTB553	ccactagtaacggccgccagtgtgctggaattctgcagatatggcgattgttgcgcaagg	oTB553-554	from pOX_twk-18[1]
	oTB554	cgggccccccctcgagcggccgccagtgtgatggatctagatgtcatgctctagat		
pNZ19/ *TWK-18 (V158D)*	oNZ52	tgtgagtggaattccaatga	oTB553-oNZ52 oTB554-oNZ53	
	oNZ53	tgctactatcctttacgcattcattggaattccactcacaGACctcagtctttactgtcttgg		
pTB314/ *TWK-18 (M280I)*	oTB571	actccaacaattggGATgaaatcgtaatcc	oTB553-oTB571 oTB554-oTB572	
	oTB572	catattgataggactcgctgtattctccacttgtgtcaat		
pOA23/ *hTASK1 WT*	oOA21	ccactagtaacggccgccagtgtgctggaattctgcagatatgaagcggcagaacgtgcg	oOA21-22	from hTASK-1-pSGEM[2]
	oOA22	agctcgggccccccctcgagcggccgccagtgtgatggattcacacggagctcctgcgct		
pOA24/ *hTASK1 (L122N)*	oOA23	ctggaacatgacATTcgtgagcgggatgcc	oOA21-23 oOA24-22	
	oOA24	ggcatcccgctcacgAATgtcatgttccag		
pOA21/ *hTASK3 WT*	oOA18	ccactagtaacggccgccagtgtgctggaattctgcagatatgaagaggcagaacgtgcg	oOA18-oIS17	from hTASK-3-pSGEM[2]
	oIS17	agctcgggccccccctcgagcggccgccagtgtgatggatctaaacggacttccggcgtt		
pOA22/ *hTASK3 (L122N)*	oOA26	gctctggaacatgacATTtgtcagcgggat	oOA18-26 oIS17-oOA25	
	oOA25	atcccgctgacaAATgtcatgttccagagc		
pOA25/ *mTASK2 WT*	oNZ56	ccactagtaacggccgccagtgtgctggaattctgcagatatggtggaccggggtccttt	oNZ56-57	from pTLN mTASK-2[3]
	oNZ57	agctcgggccccccctcgagcggccgccagtgtgatggattcacgtgcccctggggtta		
pNZ20/ *mTASK2 (L127N)*	oNZ55	gcacagcggcaccccgaaga	oNZ56-55 oNZ54-57	
	oNZ54	ctgtgtcttctacggcctcttcggggtgccgctgtgcAACacatggatcagtgccctggg		
pOA26/ *mTREK2 WT*	oOA30	ccactagtaacggccgccagtgtgctggaattctgcagatatgaaatttccaatcgagac	oOA30-31	from mTREK2c-pEXO
	oOA31	agctcgggccccccctcgagcggccgccagtgtgatggatttagtttctgtcttcaagta		
pOA27/ *mTREK2 (G196D)*	oOA43	tccagccaataagaaATCaaaaagcgggat	oOA30-43 oOA42-31	
	oOA42	atcccgctttttGATttcttattggctgga		
pOA28/ *mTRAAK WT*	oOA34	ccactagtaacggccgccagtgtgctggaattctgcagatatgcgcagcaccacactcct	oOA34-35	from mTRAAK-pEXO
	oOA35	agctcgggccccccctcgagcggccgccagtgtgatggatctacaccggcacggccttgt		
pOA29/ *mTRAAK (G133D)*	oOA45	tcccgccagcagcatATCgaacagtgggat	oOA34-45 oOA44-35	
	oOA44	atcccactgttcGATatgctgctggcggga		

pOA30/ *mTRESK WT*	oOA38	ccactagtaacggccgccagtgtgctggaa ttctgcagatatggaggctgaggagccacc	oOA38-39	from mTRESK-pEXO
	oOA39	agctcgggcccccctcgagcggccgccag tgtgatggatttaccaaggtagcgaaactt		
pOA31/ *mTRESK (F156N)*	oOA41	gtctgtgaggaccagATTcattagagggat	oOA38-41 oOA40-39	
	oOA40	atccctctaatgAATctggtcctcacagac		
	oTB			

[1] purchased from addgene
[2] kindly provided by V.K. Renigunta
[3] kindly provided by F.V. Sepúlveda
